# Temporal and spatial trend analysis of all-cause depression burden based on Global Burden of Disease (GBD) 2019 study

**DOI:** 10.1038/s41598-024-62381-9

**Published:** 2024-05-29

**Authors:** Junjiao Liu, Yueyang Liu, Wenjun Ma, Yan Tong, Jianzhong Zheng

**Affiliations:** 1 College of Public Health, Shanxi Medical University, Taiyuan, Shanxi China; 2 Second Clinical Medical College, Nanchang University, Nanchang, Jiangxi China

**Keywords:** Diseases, Health care, Medical research

## Abstract

Depression has been reported as one of the most prevalent psychiatric illnesses globally. This study aimed to obtain information on the global burden of depression and its associated spatiotemporal variation, by exploring the correlation between the global burden of depression and the social development index (SDI) and associated risk factors. Using data from the Global Burden of Disease study from 1990 to 2019, we described the prevalence and burden of disease in 204 countries across 21 regions, including sex and age differences and the relationship between the global disease burden and SDI. The age-standardized rate and estimated annual percentage change were used to assess the global burden of depression. Individuals with documented depression globally ranged from 182,183,358 in 1990 to 290,185,742 in 2019, representing an increase of 0.59%. More patients experienced major depressive disorder than dysthymia. The incidence and disability-adjusted life years of depression were the highest in the 60–64 age group and much higher in females than in males, with this trend occurring across all ages. The age-standardized incidence and adjusted life-years-disability rates varied with different SDI levels. Relevant risk factors for depression were identified. National governments must support research to improve prevention and treatment interventions.

## Introduction

Mental disorders are a major contributor to the global burden of disease. It commonly results in a higher incidence of physical decline^1–4^ and early death compared with normal age-related deaths^5^. The WHO Mental Health Action Plan 2013–2020 states that individuals suffering from mental illness have elevated rates of disability and death^6^. Individuals with depression and schizophrenia are 40–60% more likely to die suddenly compared to the general population, mainly because of undetected and untreated physical health problems they may be suffering from, such as cancer, cardiovascular disease, diabetes, and HIV infection and even suicide. Suicide is the second most common cause of death for young people in the world^7^.

There is evidence that depression predisposes individuals to myocardial infection and diabetes and having these illnesses increases the chances of developing depression. Many risk factors, such as low social status, alcohol abuse, and stress, are responsible for the development of mental illnesses^8^. Overall, mental illnesses, neurological disorders, and substance use disorders account for 13% of the global disease burden, with depression accounting for 4.3% of the total. Further, these are considered to be one of the main causes of disability worldwide, especially for female.

The economic costs of these health problems are enormous. According to a new study, the total financial costs of mental illness worldwide will reach $16.3 trillion between 2011 and 2030^9^. The Action Plan targets mental illness, stating that by 2020 countries’ suicide rates will have dropped by 10% and there will be a 20% increase in health care services for serious mental illnesses, including psychosis, bipolar disorder, and moderate-to-severe depression^10^.

Mental disorders are recognized as a major contributor to the global burden of disease, accounting for 1566.2 disability-adjusted life years (DALYs) per 100,000 of the global population in 2019. Among these, depressive disorders (major depressive disorder [MDD] and dysthymia) constituted the most significant proportion of mental disorder DALYs (37.3%)^11^. Depression can impair normal body functions and lead to depressive thoughts, which seriously affects people’s quality of life and is one of the most prevalent chronic mental illnesses worldwide. Its main symptoms are depressed emotions, declining interest, slowed thinking, sleep and eating disorders, and even suicidal thoughts in severe cases^12–14^. Around the world, in excess of 300 million people experience depression, which the WHO ranks it as the greatest contributor to global disability^15^. Most concerning of all, youth suffering from major depression have a 30 times higher likelihood of taking their own lives^16^. Despite the fact that depression have become one of the foremost health problems globally, little is known about their complex pathogenesis^17,18^.

The Global Burden of Disease (GBD) study offers detailed data on a wide range of diseases for 204 countries in 21 different regions worldwide^19^. The GBD database provides extensive information on the incidence of depression worldwide and categorizes depression into two broad groups: MDD and dysthymia^20^. We explored temporal trends and spatial distributions of depressive disorders, age and sex distributions, and the relationship between the Social Development Index (SDI) and risk factors affecting depression using data on depressive disorders in the GBD database from 1990 to 2019^20^. We described incidence and DALY rates, as well as estimated annual percentage change (EAPC) in incidence and DALY cases. The age-standardized rate (ASR) was considered to be increasing if the EAPC and the corresponding 95% CI were > 0, and the opposite were < 0. Outcomes of this study will contribute to our understanding of the global disease burden of depression, which is of great societal value in controlling and combating depression, and scientifically important for the development of psychology and neuroscience^21^.

## Methods

### Data sources

The data utilized in this study are available on the Global Health Data Exchange GBD Results Tool (http://ghdx.healthdata.org/gbd-results-tool). GBD 2019 estimated incidence, prevalence, mortality, years lived with disability (YLDs), years of life lost (YLLs), and DALYs for 369 diseases and injuries, for males and females, 23 age groups, 204 countries and territories that were geographically grouped into 21 regions from 1990 onwards^11^. All measures are presented as quantities, ratios, and percentages; they can be selected by sex, age group, and region according to the needs of the study^10^. In this study, we extracted data on depression across all age groups and sexes from 21 GBD regions and 204 countries from 1990 to 2019. As not all diseases were estimated for all countries, the GBD 2019 study used the Bayesian meta-regression tool DisMod-MR 2.1 as the principal method to ensure that the incidence, prevalence, and mortality rates for each disease were consistent^22,23^.

### Classification and definitions

#### Depressive disorders, MDD, and dysthymia

In the International Classification of Diseases Tenth Revision (ICD-10), depressive disorders were categorised into two main groups: major depressive disorder (MDD) and dysthymia. Therefore, in the GBD study, both MDD and dysthymia were included in the category of depressive disorders. MDD is an episodic depressive disorder that may recur throughout an individual’s life, with each recurrence varying in severity. Dysthymia is a slow and mild persistent depressive disorder with symptoms less severe than those of MDD, but with a course characterised by persistence. Cases that met the diagnostic criteria for MDD and dysthymia according to the DSM (Diagnostic and Statistical Manual) and ICD (Diagnostic and Statistical Manual) were included in the GBD research disease model^24^.

#### SDI

The SDI is an aggregative metric that measures the development of a country or region, combining data on the total fertility rate for females under 25, the average level of education of females aged 15 and over, and per capita income. The GBD 2019 database categorises the world into five types of regions based on the SDI index: low-SDI (0–0.45), low-middle-SDI (0.45–0.61), middle-SDI (0.61–0.69), high-middle-SDI (0.69–0.81), and high-SDI (0.81–1)^10,24^.

#### Human development index (HDI)

HDI is an aggregative indicator that measure the level of economic and social development of United Nations Member States and consists of three basic variables: life expectancy, educational attainment, and quality of life. We obtained the 2019 HDI data from the United Nations Development Program’s Human Development Report to explore the association of the HDI and the EAPC for incidence and DALYs (https://hdr.undp.org/en/composite/HDI, accessed on March 27, 2022)^25^.

#### ASR

Age-standardized rate (ASR) is a common indicator in epidemiology. When the composition of age structure is different between several comparison groups, the crude rate of direct comparison groups will lead to bias because it does not indicate whether the high incidence rate in a particular area is due to differences in age composition, and it is usually necessary to compare rates after standardization^26^. Therefore, the age-standardized incidence rate reflects the incidence level that is not affected by age factors, and does not represent the absolute incidence rate, but only to facilitate the comparison of incidence data in different regions or different periods.

The age-standardized rate was calculated on the basis of the following formula:

$$\text{Age standardized rate}=\frac{{\sum }_{i=1}^{A}{a}_{i}{W}_{i}}{{\sum }_{i=1}^{A}{w}_{i}}\times \text{100,000}$$

The age-standardized rate per 100,000 population is equal to the sum of the products of age-specific rates (wi, where* i* denotes the *i*th age class) and number of cases (or weight; wi) in the same age subgroup *i* of the selected reference standard population and then divided by the sum of the standard population weights^27^. Age-standardized rates were calculated considering the GBD world population. In this study, ASR was used to quantify the incidence of two types of depression and the trend of DALYs^28^.

#### EAPC

The EAPC provides a well-recognized approach of characterizing ASR using a regression model that quantifies the average annual rate of change during a specific period, with the plus and minus signs representing the direction of change. The regression line was used to estimate the natural logarithm of the rate (i.e., y = α + βx + ϵ, where y = ln(ASR) and x = calendar year). The EAPC was calculated as 100 × (exp (β) − 1), with a 95% CI derived from a linear regression model. All statistics were analysed using R version 4.2.3, and a two-sided P < 0.05 was considered statistically significant^28^. The ASR was decreasing when both the EAPC and the upper limit of its 95% CI were ≤ 0; conversely, the ASR was increasing when both were ≥ 0; otherwise the ASR was stable. All statistical analyses were made with R software^19^.

### Analytic strategy

We depicted changes in the prevalence and burden of disease of depression in 204 nations covering 21 distinct regions during the study period. The analysis indices included incidence and DALYs. The ASR was calculated considering the average population structure of the world from 2000 to 2025 as the standard population structure^25^. DALYs = YLDs + YLLs. Because depressive disorders are non-fatal diseases, YLDs are equivalent to DALYs in this instance. This study highlights the state of the burden of disease for depression and temporal and spatial trends from 1990 to 2019^24^. First, the incidence and DALYs of depressive disorders worldwide since 1990 to 2019 were odellin. The burden of depression was then evaluated according to age, sex, country, and region. The extent to which differing development levels affected the burden of depressive disorders was then assessed using the SDI and risk factors for depression^10^. In the result, we present 95% UI for every metric based on the 25th and 975th ordered values of 1000 draws of the posterior distribution. Uncertainty interval (UI) is the interval estimated by GBD research calculation method DisMod-MR, a Bayesian meta-regression tool. It takes into account the differences between different calculation methods in different countries, as well as the uncertainty of multiple filling of missing data values in different countries, which is obtained by repeated sampling calculation through the correlation matrix. Unlike confidence intervals, the UI not only adjusts for sampling error, but also captures uncertainty in multiple stages of analysis modelling and adjusts for the type and quality of data sources^28^.

### Ethical committee

The study was compliant with the Guidelines for Accurate and Transparent Health Estimates Reporting, and the University of Washington Institutional Review Board reviewed and approved the waiver of informed consent for GBD 2019.

## Results

**Figure 1:**
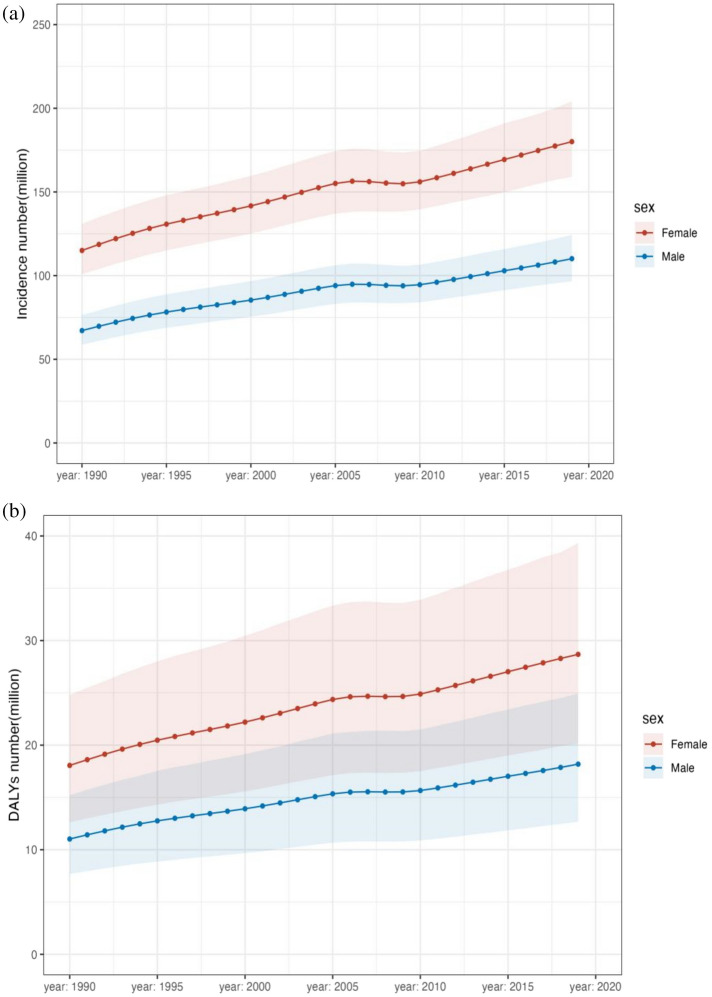
Temporal trend of global incidence (**a**) and DALYs (**b**) number of depressive disorders.

**Figure 2:**
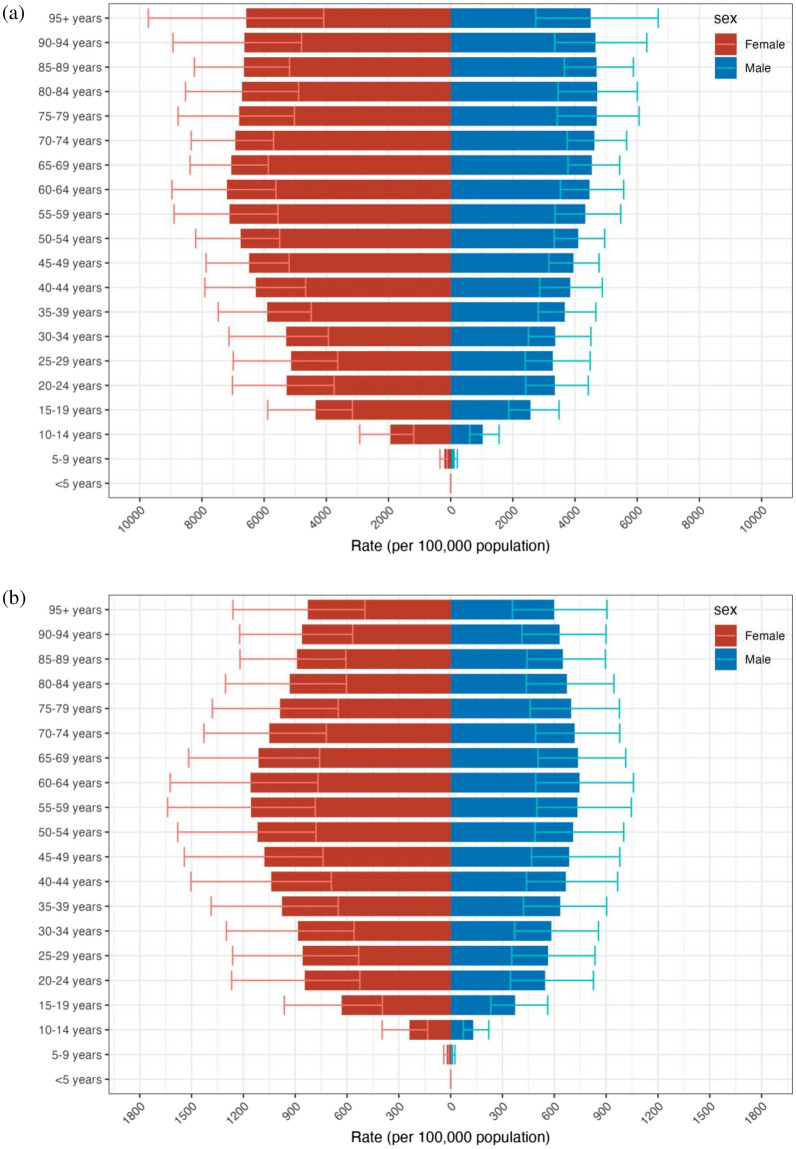
Age-standardized incidence rate (**a**) and age-standardized DALYs rate (**b**) trends of sex and age distribution.

Since 1990 to 2019, depressive disorder cases have grown from 182,183,358 (95% UI 159,598,111–207,533,227) to 290,185,742 (95% UI 256,024,052–328,260,553), with a 0.59% (95% UI 0.55–0.64) increase in cases of depression for both male and female (see Supplementary Table S1a). In 2019, 46,863,642 (95% UI 32,929,363–63,797,315) DALYs resulting from depression were documented with an upward trend of 0.61% since 1990 (see Table 1, Supplementary Table S1b). The incidence of depression was greater among females than males from 1990 to 2019, as were associated DALYs (see Fig. 1, Table 1). In 2019, depression caused 110,123,422 (95% UI 96,668,365–124,305,433) incidence cases in males globally, and 180,062,320 (95% UI 159,076,846–204,131,417) incidence cases in females, resulting in 18,183,102 (95% UI 12,682,047–24,947,035) DALYs in males, and 28,680,540 (95% UI 20,155,773–39,319,358) DALYs in females. The age-standardised incidence rate (ASIR) of depressive disorders is found to grow with age, reaching a peak during the 60–64 year age group for females and the 80–84 year age group for males. However, there was a small decrease in the 25–29 years age group across both sexes. The age-standardized adjusted life-year disability rate (ASDR) of depression also showed an increasing trend with age. It began to decline in females after reaching a peak in the 55–59 age group. And for males, it peaks in the 60–64 age group and then begins to decline. ASDR for males and females were 452.17 (95% UI 316.79–618.13) and 702.08 (95% UI 492.3–963.58), respectively (see Fig. 2, Table 1). Over all, Females develop a greater ASIR and ASDR than males in the same age group. However, during the period 1990–2019, the DALYs change was higher in males than in females, 0.65% in males (95% UI 0.61–0.69) compared to 0.59% in females (95% UI 0.54–0.63) (see Supplementary Table S1b).

**Table 1:** Global burden of depressive disorder in 2019 for both sexes and 27 regions, with EAPC from 1990 and 2019.

Characteristics	Incidence (95% UI)			DALYs (95% UI)		
	Number	ASR	EAPC	Number	ASR	EAPC
Global	290,185,742 (256,024,052–328,260,553)	3588.25 (3152.71–4060.42)	− 0.29% (− 0.38 to − 0.21)	46,863,642 (32,929,363–63,797,315)	577.75 (405.79–788.88)	− 0.24% (− 0.31 to − 0.16)
Sex						
Male	110,123,422 (96,668,365–124,305,433)	2750.27 (2419.66–3104.07)	− 0.21% (− 0.3 to − 0.11)	18,183,102 (12,682,047–24,947,035)	452.17 (316.79–618.13)	− 0.17% (− 0.25 to − 0.09)
Female	180,062,320 (159,076,846–204,131,417)	4416.34 (3886.9–5015.49)	− 0.35% (− 0.43 to − 0.26)	28,680,540 (20,155,773–39,319,358)	702.08 (492.3–963.58)	− 0.28% (− 0.35 to − 0.21)
Category						
Depressive disorders	290,185,742 (256,024,052–328,260,553)	3588.25 (3152.71–4060.42)	− 0.29% (− 0.38 to − 0.21)	46,863,642 (32,929,363–63,797,315)	577.75 (405.79–788.88)	− 0.24% (− 0.31 to − 0.16)
Major depressive disorder	274,803,790 (241,280,545–312,774,423)	3397.48 (2978.66–3866.97)	− 0.31% (− 0.41 to − 0.22)	37,202,742 (25,650,205–51,217,042)	459.59 (315.19–634.72)	− 0.32% (− 0.41 to − 0.22)
Dysthymia	15,381,951 (12,782,128–18,474,451)	190.77 (158.69–229.44)	0.08% (0.07–0.09)	9,660,901 (6,311,566–14,421,787)	118.16 (77.31–176.65)	0.09% (0.08–0.1)
Socio− demographic index						
High SDI	44,711,792 (39,796,761–50,166,003)	4013.63 (3545.48–4550.43)	0.31% (0.18–0.44)	7,025,129 (4,955,200–9,506,636)	626.84 (438.47–852.48)	0.23% (0.14–0.33)
High− middle SDI	53,642,569 (47,529,706–60,307,945)	3184.21 (2809.6–3583.66)	− 0.5% (− 0.57 to − 0.43)	8,896,917 (6,247,986–12,123,142)	523.01 (367.02–713.05)	− 0.4% (− 0.46 to − 0.34)
Middle SDI	80,760,069 (71,066,732–91,500,542)	3139 (2765.35–3540.43)	− 0.2% (− 0.28 to − 0.13)	13,541,947 (9,515,935–18,454,507)	521.68 (366.8–709.93)	− 0.18% (− 0.24 to − 0.13)
Low-middle SDI	70,155,480 (61,292,237–79,973,480)	4180.3 (3660.97–4740.48)	− 0.62% (− 0.79 to − 0.44)	11,026,538 (7,715,898–15,191,253)	654.34 (458.32–897.85)	− 0.51% (− 0.66 to − 0.36)
Low SDI	40,743,981 (34,959,157–47,317,678)	4770.22 (4142.24–5461.66)	− 0.38% (− 0.5 to − 0.26)	6,345,789 (4,316,623–8,788,145)	738.87 (514.68–1011.24)	− 0.3% (− 0.4 to − 0.2)
Region						
Andean Latin America	1,809,802 (1,561,707–2,084,373)	2886.58 (2499.39–3315.19)	− 0.29% (− 0.34 to − 0.25)	290,671 (198,567–403,423)	462.07 (318.12–640.03)	− 0.25% (− 0.29 to − 0.21)
Australasia	1,539,866 (1,329,848–1,771,752)	5079.18 (4368.05–5925.8)	0.07% (− 0.06–0.21)	237,564 (164,169–330,311)	777.82 (538.62–1094.07)	0.07% (− 0.05–0.2)
Caribbean	2,156,062 (1,859,121–2,484,561)	4336.17 (3737.42–5007.13)	− 0.51% (− 0.56 to − 0.45)	327,025 (226,095–450,595)	657.19 (454.07–905.93)	− 0.49% (− 0.54 to − 0.43)
Central Asia	2,980,970 (2,577,906–3,464,935)	3327.42 (2888.66–3825.36)	− 0.19% (− 0.21 to − 0.16)	486,600 (334,518–679,840)	534.9 (372.57–741.82)	− 0.16% (− 0.18 to − 0.13)
Central Europe	3,557,074 (3,134,283–4,043,451)	2436.8 (2132.45–2771.69)	− 0.67% (− 0.74 to − 0.59)	596,440 (420,305–816,082)	413.89 (290.54–572.31)	− 0.54% (− 0.6 to − 0.48)
Central Latin America	9,412,732 (8,221,932–10,719,231)	3675.78 (3219.65–4181.96)	0.34% (0.3–0.37)	1,447,181 (1,009,408–1,981,151)	563.62 (392.72–771.17)	0.31% (0.28–0.34)
Central Sub-Saharan Africa	6,714,339 (5,590,521–8,062,124)	6646.94 (5680.5–7819.84)	− 0.17% (− 0.18 to − 0.15)	1,010,267 (681,633–1,430,655)	1000.16 (682.15–1397.69)	− 0.12% (− 0.14 to − 0.11)
East Asia	42,235,926 (37,513,979–47,555,038)	2292.26 (2043.67–2562.45)	− 0.8% (− 0.97 to − 0.64)	7,802,555 (5,472,939–10,767,864)	415.98 (291.93–573.46)	− 0.67% (− 0.78 to − 0.56)
Eastern Europe	9,150,637 (7,960,749–10,432,658)	3546.8 (3076.08–4062.82)	− 0.57% (− 0.66 to − 0.47)	1,442,695 (1,013,990–1,986,605)	562.24 (391.45–771.76)	− 0.46% (− 0.54 to − 0.38)
Eastern Sub-Saharan Africa	16,013,047 (13,725,944–18,554,113)	5466.48 (4781.02–6234.48)	− 0.32% (− 0.39 to − 0.26)	2,510,165 (1,702,208–3,475,451)	845.4 (589.89–1154.93)	− 0.26% (− 0.32 to − 0.21)
High-income Asia Pacific	5,193,652 (4,652,118–5,735,090)	2320.99 (2063.54–2600.8)	0.4% (0.27–0.53)	812,255 (572,741–1,104,805)	365.65 (253.57–499.26)	0.31% (0.2–0.43)
High-income North America	18,459,876 (16,429,393–20,674,357)	4885.16 (4308.48–5532.44)	0.62% (0.32–0.92)	2,864,089 (2,023,936–3,872,481)	753.77 (525.53–1023.69)	0.43% (0.2–0.66)
North Africa and Middle East	31,006,695 (26,270,019–36,438,429)	5098.6 (4378.86–5947.72)	0.06% (0.03–0.09)	4,767,774 (3,261,470–6,600,677)	781.06 (535.18–1075.62)	0.06% (0.03–0.08)
Oceania	328,505 (274,947–393,381)	2711.59 (2306.26–3193.17)	− 0.16% (− 0.17 to − 0.15)	56,577 (38,501–80,206)	476.09 (325.58–663.22)	− 0.13% (− 0.13 to − 0.12)
South Asia	71,998,403 (62,917,271–81,675,123)	4179.15 (3668.72–4727.18)	− 0.85% (− 1.1 to − 0.6)	11,188,435 (7,828,808–15,283,076)	645.08 (452.66–877.7)	− 0.71% (− 0.92 to − 0.5)
Southeast Asia	14,451,056 (12,506,180–16,471,186)	2060.52 (1797.73–2341)	− 0.19% (− 0.25 to − 0.14)	2,753,223 (1,898,460–3,795,437)	389.23 (270.38–536.55)	− 0.13% (− 0.16 to − 0.09)
Southern Latin America	2,362,146 (2,089,297–2,658,887)	3313.55 (2925.62–3745.45)	− 0.42% (− 0.5 to − 0.34)	359,571 (249,695–491,681)	503.29 (349.65–690.9)	− 0.42% (− 0.5 to − 0.34)
Southern Sub-Saharan Africa	3,344,012 (2,915,269–3,791,826)	4552.32 (4015.91–5105.97)	0.13% (0.03–0.24)	524,604 (368,831–719,717)	705.61 (497.87–958.57)	0.09% (− 0.01–0.19)
Tropical Latin America	10,928,342 (9,746,995–12,123,340)	4560.16 (4084.43–5058)	− 0.32% (− 0.62 to − 0.02)	1,652,267 (1,159,774–2,244,114)	686.08 (482.44–932.46)	− 0.27% (− 0.54–0.01)
Western Europe	22,312,186 (19,873,592–25,015,077)	4347.46 (3841.95–4912.74)	− 0.11% (− 0.14 to − 0.08)	3,463,005 (2,438,349–4,706,017)	677.2 (475.01–929.5)	− 0.09% (− 0.11 to − 0.06)
Western Sub-Saharan Africa	14,230,414 (12,217,181–16,431,648)	4407.3 (3851.35–5021.82)	− 0.25% (− 0.43 to − 0.07)	2,270,679 (1,552,645–3,123,065)	693.84 (485.18–949.29)	− 0.2% (− 0.36 to − 0.04)

In terms of the subtypes of depression, the incidence of MDD was much more prevalent than dysthymia at 2,784,803,790 (95% UI 241,280,545–312,774,423) and 15,381,951(95% UI 12,782,128–18,474,451) respectively in 2019. The same was true for DALYs, which were 37,202,742 (95% UI 25,650,205–51,217,042) and 9,660,901 (95% UI 6,311,566–14,421,787) respectively (see Table 1). During the period 1990–2019, the global depression ASIR has decreased significantly (EAPC =  − 0.29%, 95% UI  − 0.38 to − 0.21), whereas the ASDR has markedly increased (EAPC = 0.61%, 95% UI 0.57–0.65) (see Table 1).

### Global burden and EAPC of depressive disorders by 21 GBD regions

Individuals with depressive disorders increased in all five SDI regions from 1990 through 2019 (see Supplementary Table S1a). However, the ASIR decreased in the high-middle-SDI (EAPC =  − 0.5%, 95% UI  − 0.57 to − 0.43), middle-SDI (EAPC =  − 0.2%, 95% UI  − 0.28 to − 0.13), low-middle-SDI (EAPC =  − 0.62%, 95% UI  − 0.79 to − 0.44), and low-SDI regions (EAPC =  − 0.38%, 95% UI  − 0.5 to − 0.26), only increasing in the high-SDI regions (EAPC = 0.31%, 95% UI 0.18–0.44) (see Table 1). The same is true for DALYs cases and ASDR (Table 1, Supplementary Table S1b). ASDR decreased in the high-middle-SDI (EAPC =  − 0.4%, 95% UI  − 0.46 to − 0.34), middle-SDI (EAPC =  − 0.18%, 95% UI  − 0.24 to − 0.13), low-middle-SDI (EAPC =  − 0.51%, 95% UI − 0.66 to − 0.36) and low-SDI regions (EAPC =  − 0.3%, 95% UI  − 0.4 to − 0.2), only increasing in the high-SDI regions (EAPC = 0.23%, 95% UI 0.14–0.33) (see Table 1, Supplementary Table S1b).

**Figure 3:**
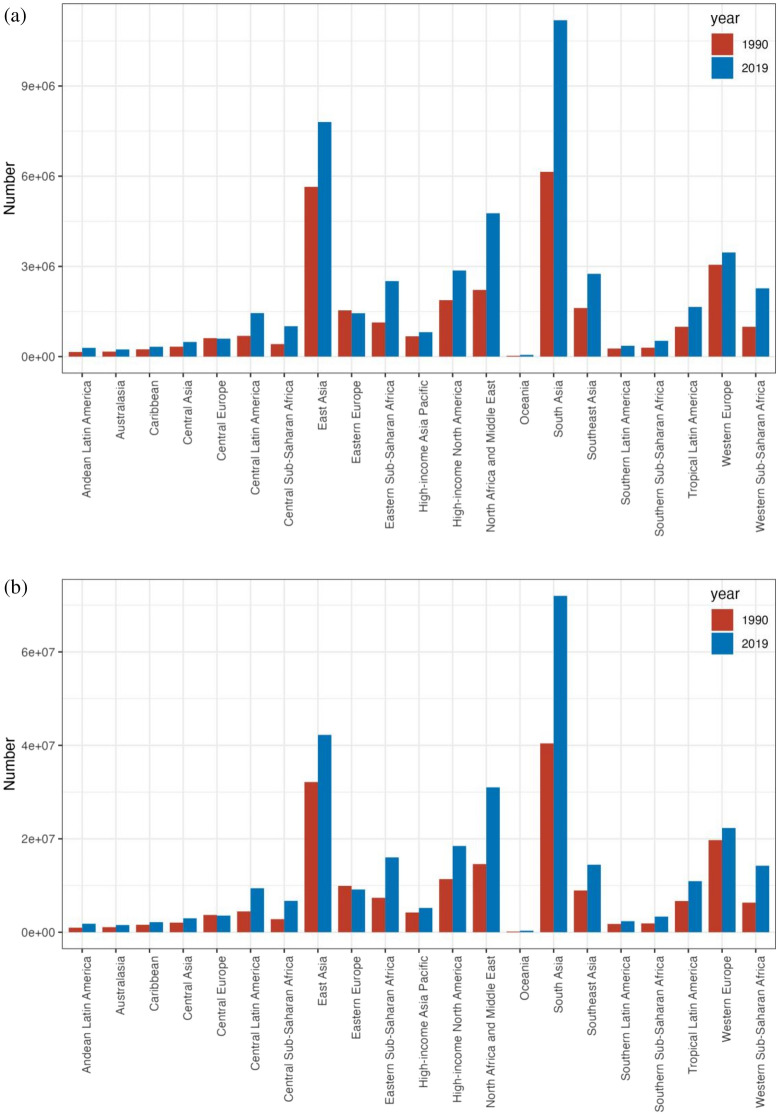
The incident cases (**a**) and DALYs (**b**) of depression at a regional level. The left column in each group is case data in 1990 and the right column in 2019.

The incidence of depressive disorders grew in all regions, with a decline only in Central and Eastern Europe (see Fig. 3a). Central Sub-Saharan Africa saw the maximum rate of increase (1.4%, 95% UI 1.29–1.51), followed by Western Sub-Saharan Africa (1.25%, 95% UI 1.22–1.28) and Eastern Sub-Saharan Africa (1.17%, 95% UI 1.13–1.21), with the decline being most marked in Eastern Europe (− 0.08%, 95% UI  − 0.1 to − 0.05) (see Supplementary Table S1a). There was a marked rise in ASR across high-income North America (EAPC = 0.62, 95% UI 0.32–0.92) and a marked drop in South Asia (EAPC =  − 0.85, 95% UI  − 1.1 to − 0.6) (see Table 1).

DALYs corresponding to depressive disorders grown in all geographical regions, with a decline only in Central and Eastern Europe (see Fig. 3b). The largest increase was occurred in Central Sub-Saharan Africa (1.43%, 95% UI 1.33–1.54), followed by Western Sub-Saharan Africa (1.29%, 95% UI 1.26–1.32) and Eastern Sub-Saharan Africa (1.21%, 95% UI 1.17–1.25), with the decline being most marked in Eastern Europe (− 0.06%, 95% UI − 0.08 to − 0.04) (Supplementary Table S1b). There was a marked rise in ASR in Central Sub-Saharan Africa (EAPC = 1.43, 95% UI 1.33–1.54) and a decline in South Asia (EAPC =  − 0.71, 95% UI  − 0.92 to − 0.5) (see Table 1).

### Global burden and EAPC of depressive disorders across 204 countries and territories

**Figure 4:**
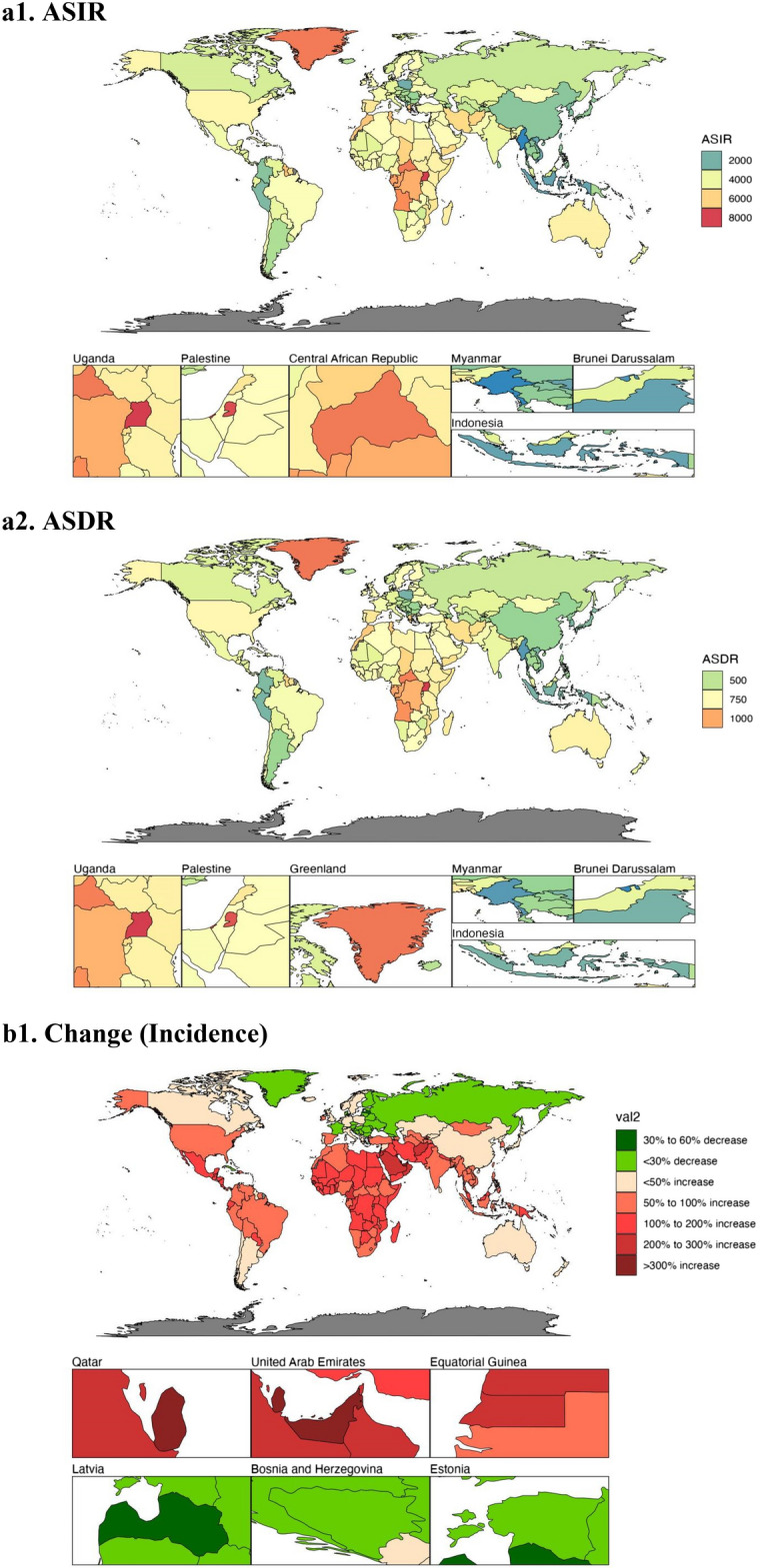

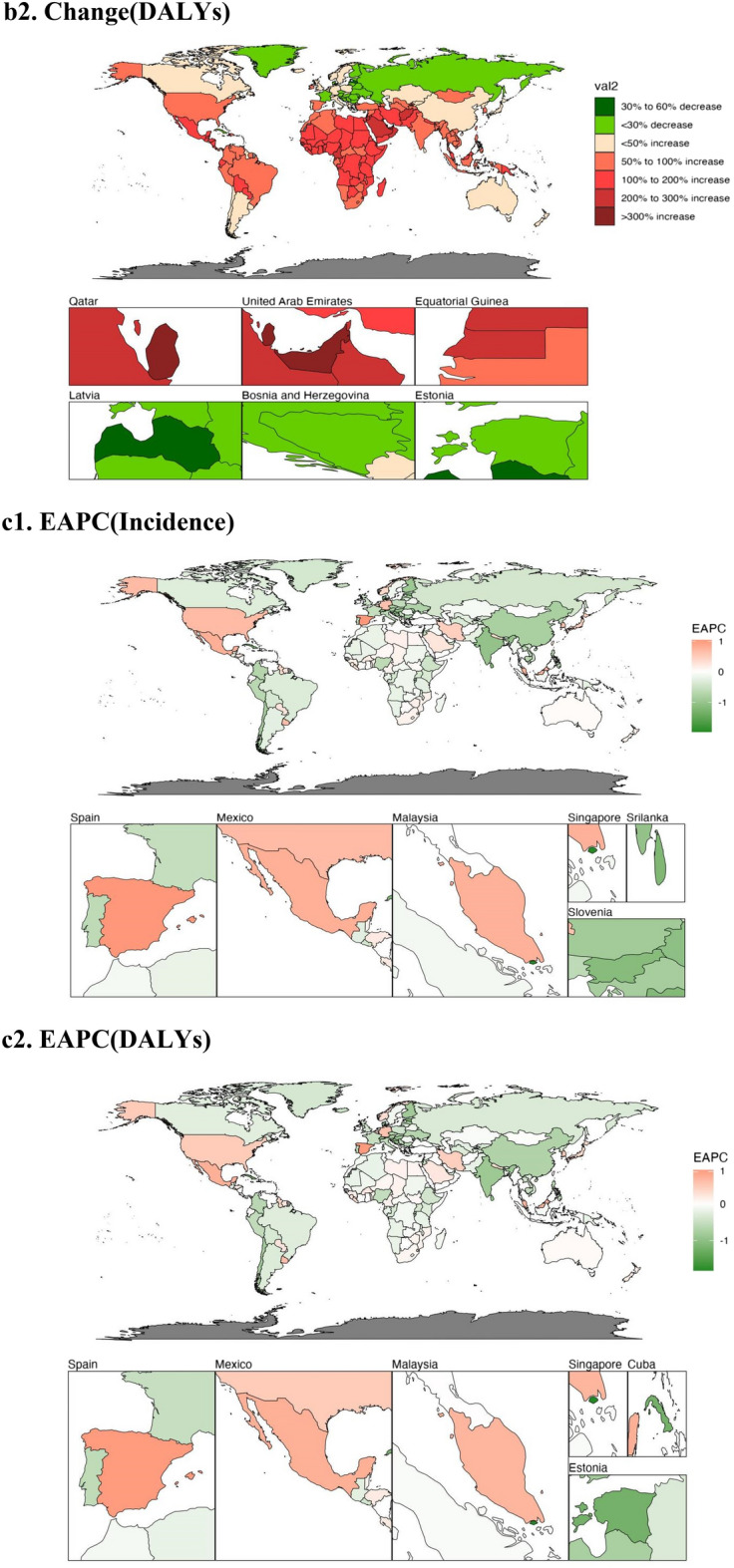
The global disease burden of depression for both sexes in 204 countries and territories. (**a1**) The ASIR of depression in 2019; (**a2**) the ASDR of depression in 2019; (**b1**) the relative change in incident cases of depression between 1990 and 2019; (**b2**) the relative change in DALYs number of depression between 1990 and 2019; (**c1**) the EAPC of depression ASIR from 1990 to 2019; (**c2**) the EAPC of depression ASDR from 1990 to 2019. *ASIR* age-standardized incidence rate, *ASDR* age-standardized DALYs rate, *EAPC* estimated annual percentage change (Image generated in R software version 4.2.3 (https://cran.r-project.org)).

The ASIR for depression varied dramatically across 204 countries and territories in 2019 (see Fig. 4a, Supplementary Table S2a). The ASIR was highest in Uganda (8062.76, 95% UI 6946.5–9436.97), followed by Palestine (7864.2, 95% UI 6719.71–9216.83) and the Central African Republic (7230.55, 95% UI 6121.98–8465.86), and was lowest in Myanmar (1393.92, 95% UI 1188.1–1612.65), followed by Brunei Darussalam (1,575.58, 95% UI 1346.16 to 1854.56) and Indonesia (1794.07, 95% UI 1557.18–2060.02). Of the 204 countries and territories under analysed, the ASDR for depressive disorders in 2019 differed considerably (see Fig. 4a, Supplementary Table S2b), with the highest being in Uganda (1212.09, 95% UI 824.6–1696.26), followed by Palestine (1168.68, 95% UI 802.95–1624.31) and Greenland (1098.69, 95% UI 750.76–1540.82), and the lowest in Brunei Darussalam (260.29, 95% UI 177.62–360.71), followed by Myanmar (298.19, 95% UI 203.44–421.71) and Indonesia (350.26, 95% UI–242.89–488.45).

The global incidence of depression grew by 0.59% from 182,183,358 in 1990 to 290,185,742 in 2019. Qatar had the greatest growth (5.89%, 95% UI 5.34–6.51), followed by the United Arab Emirates (4.81%, 95% UI 4.09–5.53) and Equatorial Guinea (2.42%, 95% UI 2.19–2.67) (see Fig. 4b, Supplementary Table S2a). From 1990 to 2019, depression declined in 23 nations, with Latvia experiencing the largest reduction (− 0.32%, 95% UI  − 0.38 to − 0.24), followed by Bosnia and Herzegovina (− 0.3%, 95% UI − 0.38 to − 0.21) and Estonia (− 0.27%, 95% UI − 0.34 to − 0.19). The DALYs for depression worldwide rose from 29,089,267 in 1990 to 46,863,642 in 2019, which is a 0.61% increase. Qatar had the most significant growth (5.99%, 95% UI 5.45–6.57), followed by the United Arab Emirates (4.91%, 95% UI 4.24 to 5.65) and Equatorial Guinea (2.48%, 95% UI 2.26 to 2.73) (see Fig. 4b, Supplementary Table S2b). DALYs for depression declined in 21 countries, with Latvia experiencing the largest decline from 1990 to 2019 (− 0.31%, 95% UI − 0.37 to − 0.24), followed by Bosnia and Herzegovina (− 0.29%, 95% UI − 0.36 to − 0.21) and Estonia (− 0.26%, 95% UI − 0.33 to − 0.19).

Among the 204 countries and territories, the greatest rise of ASIR occurred in Spain (EAPC = 1.05, 95% UI 0.78–1.31), next is Mexico (EAPC = 0.81, 95% UI 0.73 to 0.89) and Malaysia (EAPC = 0.79, 95% UI 0.59–1.00) (see Fig. 4c, Supplementary Table S2a). The most significant drop in ASR was Singapore (EAPC =  − 1.97, 95% UI − 2.22 to − 1.73), then Sri Lanka (EAPC =  − 1.23, 95% UI − 1.4 to − 1.06) and Slovenia (EAPC =  − 1.15, 95% UI − 1.23 to − 1.07). Among all 204 countries and territories, Spain had the greatest rise in ASDR (EAPC = 0.94, 95% UI 0.69–1.18), then Mexico (EAPC = 0.74, 95% UI 0.67–0.81) and Malaysia (EAPC = 0.69, 95% UI 0.53–0.86) (see Fig. 4c, Supplementary Table S2b). The greatest decline in ASR was in Singapore (EAPC =  − 1.83, 95% UI − 2.06 to − 1.59), next is Cuba (EAPC =  − 1.23, 95% UI − 1.36 to − 1.1) and Estonia (EAPC =  − 1.23, 95% UI − 1.36 to − 1.1).

### The correlation of SDI with the global burden of depressive disorders

**Figure 5:**
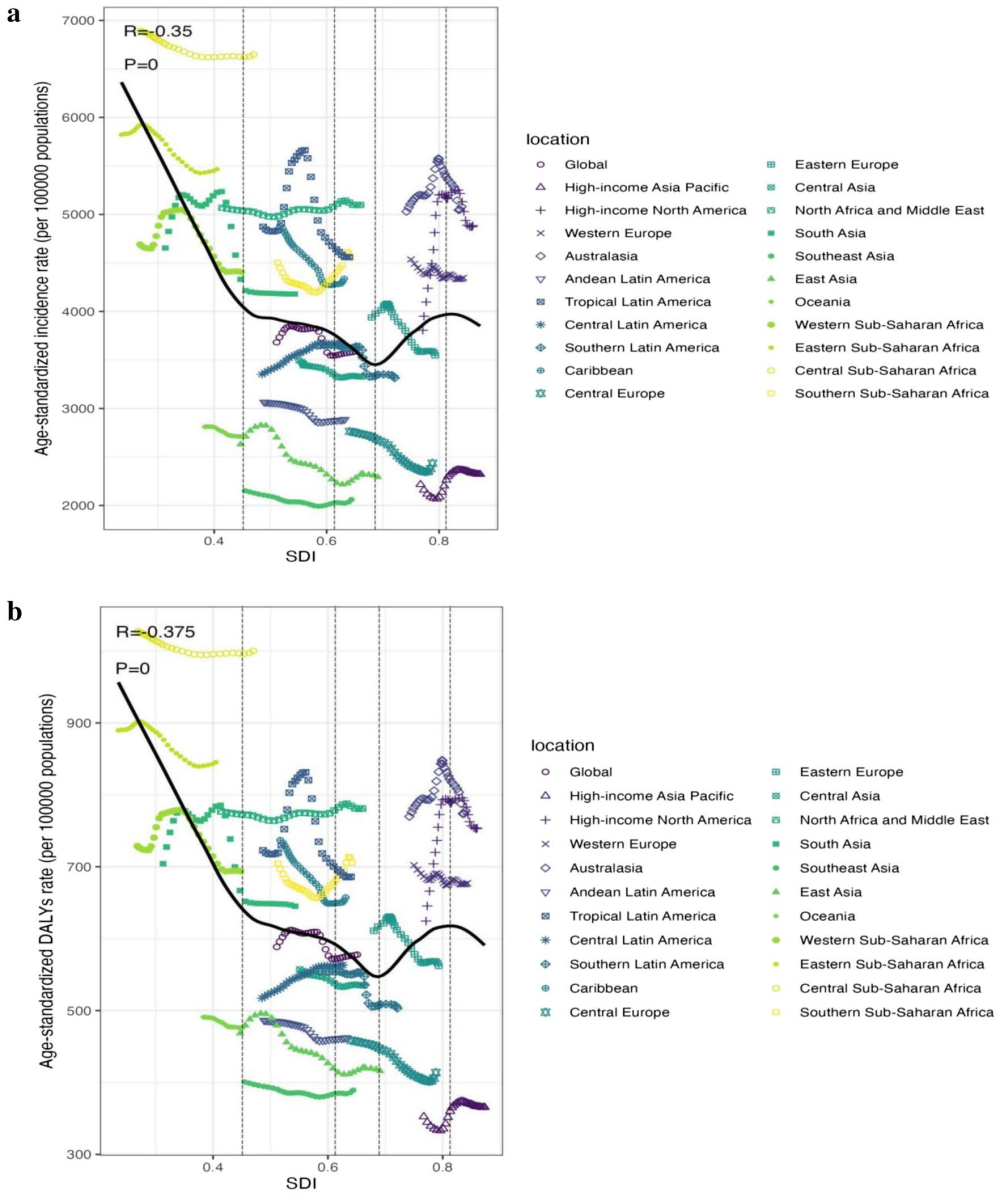

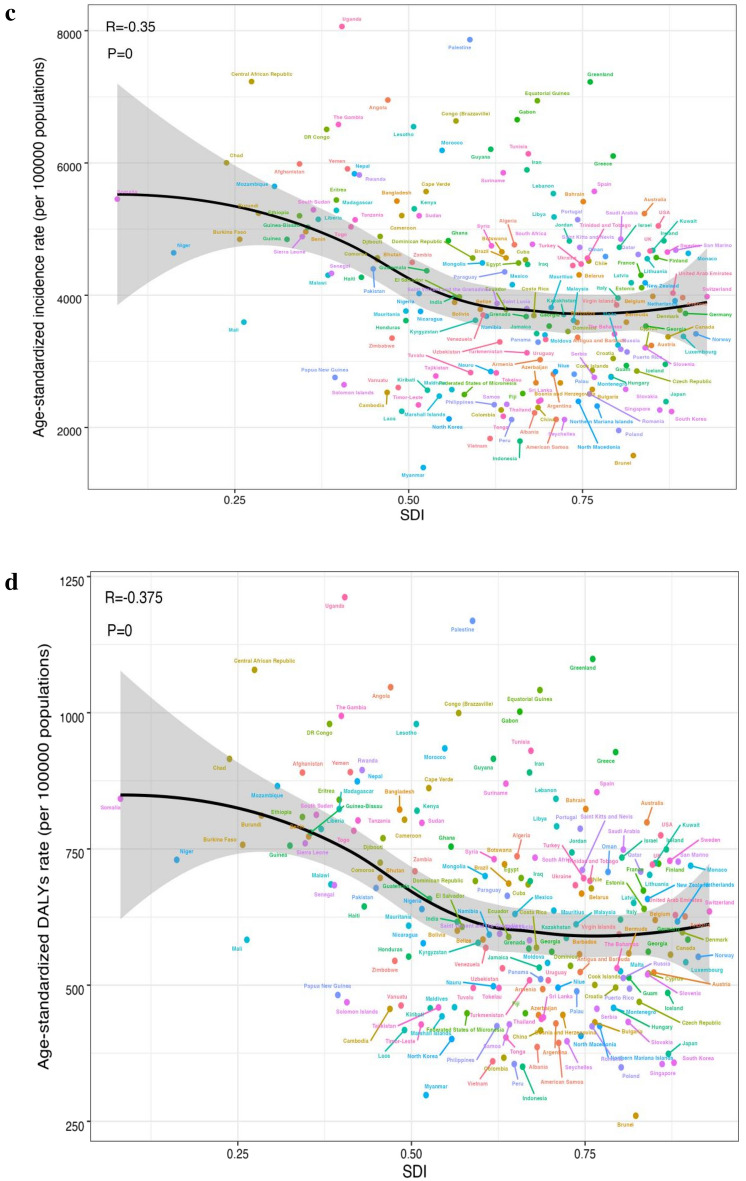
Age-standardized incidence rate (**a**) and age-standardized DALYs rate (**b**) for depression for 21GBD regions and 204 countries and territories (**c**,**d**) by Socio-demographic Index(SDI),1990–2019 (the black line indicates the correlation between all SDI regions and the incidence rate or DALY expected value).

Substantial correlation was observed among the SDI and depression prevalence and also among the SDI and DALYs, as illustrated in Fig. 5. A number of regions exceeded the expected levels of prevalence, including Central Sub-Saharan Africa and Australasia, while a number of regions fell below the expected levels of prevalence, including South-East Asia and the high-income regions Asia and the Pacific (see Fig. 5a).

Of the 204 countries and territories whose association with the 2019 SDI was recognised, most had a negative association with the SDI, with a few countries significantly above or below the expected level. Uganda and Palestine were significantly higher than expected, while Myanmar and Brunei were significantly lower than expected (see Fig. 5c).

DALYs declined in many areas as the SDI became higher, with the exception of certain regions. For instance, the DALYs rate in Western Sub-Saharan Africa fell briefly, then rose, and then kept falling, forming an inverted U-curve. The DALYs rate of Tropical Latin America, which has a low-middle SDI rank, remained stable at first, then increased, before declining sharply. The rate of Southern Latin America, which has a middle SDI rank, remained stable at first, then decreased, and then continued to remain stable. The DALYs rate of Eastern Europe, which has a high-middle SDI, rose slightly, then fell sharply, remained stable for a period, and then fell slightly. High SDI ratios in high-income Asia–Pacific regions fell briefly and then rose, before falling slightly (see Fig. 5b).

Since 1990 to 2019, the DALY rates obtained in high SDI-ranking regions, such as Western Europe, were mostly consistent with expectations. However, during the study period, some regions (e.g., high-income Asia–Pacific) continued to have DALYs far lower than expected, while others (e.g., Australasia and high-income North America) continued to have DALYs higher than expected (see Fig. 5b). At the country level during 2019, following a similar pattern to the association of morbidity and SDI, there was a marked adverse correlation between DALYs and SDI, with a few exceptions (R =  − 0.375, p < 0.001) (see Fig. 5d)^28^.

### The relationship between the HDI and the global burden of depressive disorders

**Figure 6:**
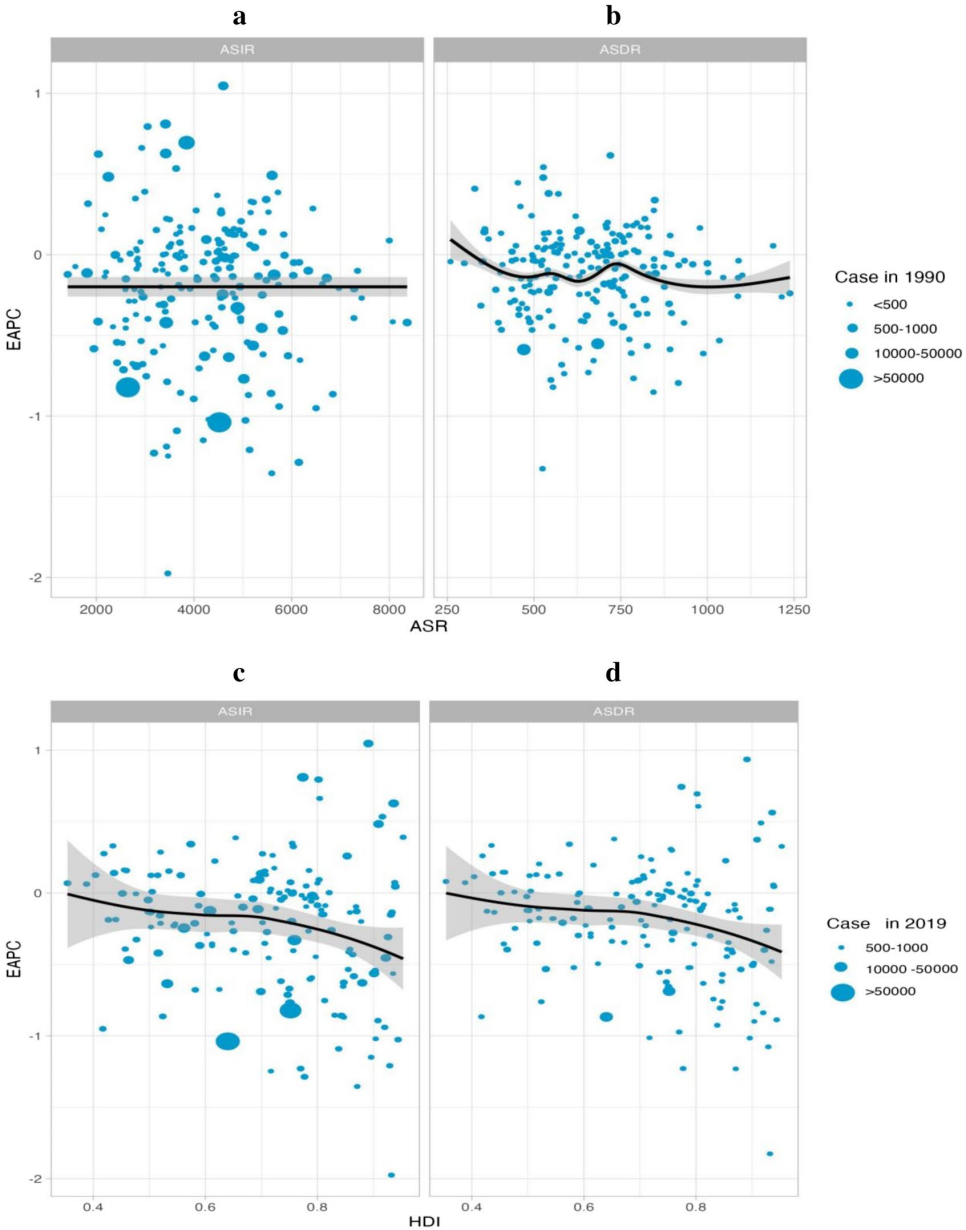
The EAPCs of depressive disorders at global, regional and national level. (**a**) The correlation between EAPC and age-standardized rate of depressive disorders incidence and (**b**) DALYs rate in 1990. (**c**,**d**) The correlation between EAPC and HDI in 2019. The circles represent countries that were available on HDI data. The size of circle is increased with the cases of depressive disorders cases. The ρ indices and p values presented were derived from Pearson correlation analysis. (**a**) ρ = − 0.064, p = 0.363 (**b**) ρ = − 0.057, p = 0.014 (**c**) ρ = − 0.198, p = 0.013 (**d**) ρ = − 0.213, p = 0.007.

No significant relationship was found between the EAPC for 1990 morbidity and morbidity (ρ =  − 0.064, p = 0.363). An inverse association was identified with EAPC in DALYs and DALYs in 1990 (ρ =  − 0.057, p = 0.014) (see Fig. 6a). In view of the fact that the EAPC was below zero, the number of DALYs attributable to depressive disorders decreased more rapidly in countries with higher DALYs in 1990. In addition, a significant negative correlation was identified for EAPC with the 2019 HDI, suggesting that prevalence rates declined more rapidly in areas with higher HDI. The same association was identified with DALYs rate and HDI (ρ =  − 0.213, p = 0.007) (see Fig. 6b).

### Risk factors of depressive disorders

**Figure 7:**
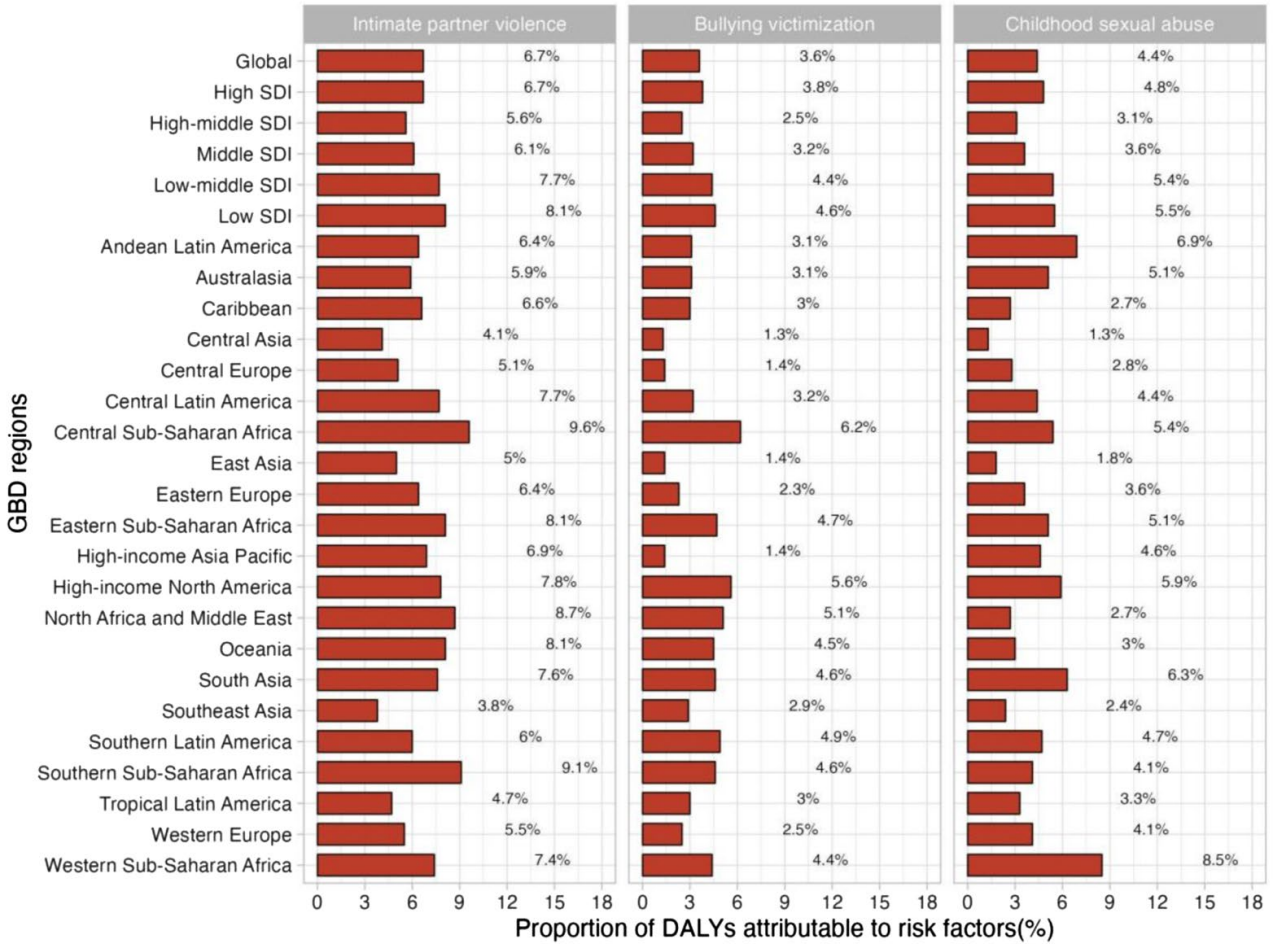
Proportion of depressive disorders DALYs attributable to intimate partner violence, bullying victimization, and childhood sexual abuse, for 21 GBD regions, 2019.

For the world as a whole, a small fraction of DALYs were ascribed to the three risk factors for which GBD estimates were obtainable, of which 6.7% attributable to intimate partner violence, 3.6% to bullying victimisation, and 4.4% to childhood sexual abuse (see Fig. 7). There is regional variation in the contributions of these risk factors. For instance, intimate partner violence had the greatest impact in Central Sub-Saharan Africa (9.6% of DALYs were attributable to intimate partner violence) and Southern Sub-Saharan Africa (9.1%), where intimate partner violence remains prevalent, and is lowest in Southeast Asia (3.8%). As well, the contribution of bullying vicitimisation was greatest in Central Sub-Saharan Africa (6.2% of DALYs attributable to bullying victimisation) and high-income North America (5.6%), and was lowest in Central Asia, where bullying victimisation is relatively low. In addition, the impact of childhood sexual abuse was greatest in Western Sub-Saharan Africa (8.5% of DALYs were attributable to childhood sexual abuse) and lowest in Central Asia. However, given that there were only three risk factors relevant to depressive disorders among the GBD study, the percentage of DALYs resulting from these three risk factors remains small when viewed as a whole, which means that there is a need for further study on the key influences of depression.

## Discussion

Depression, as a serious public health problem, is associated with adverse health outcomes and reduced life-expectancy^30^. This study presents the global burden of depression through GBD data, focusing on the temporal trends and spatial distribution of depression from 1990 to 2019, with a specific focus on EAPC. The outcome of the study offers an important reference value for all regional governments when formulating relevant prevention and treatment measures for depression^19^.

The outcomes of this study suggest that the overall burden of depression has increased rapidly within three decades, but the increase has not been uniform across age groups, sexes, or regions^10^. The further increase in the burden of depression observed in our study is in agreement with the outcomes by Liu et al.^29^. This is likely driven by the increasing social pressure brought about by economic development and population growth, and the serious aging of the population. In addition, with the economic development and the gradual improvement of people’s living standards, the demand for medical services is also rising, especially the increasing emphasis on mental health, in this case, the search for mental health services has also increased. It is also important to note that the continuous improvement of depression screening tools has made it possible for medical institutions and government agencies to obtain more comprehensive and accurate data. Our research suggests that MDD accounts for a large proportion of depression cases and is the most prevalent psychiatric category of depression, a finding which aligns with those of a 2022 study by Li et al.^31^.

Our research shows that females have higher rates of depression and DALYs than males across all age groups. The prevalence and DALYs rates of depression were highest in people aged 60–64 years. There are many reasons for this. The psychological characteristics of people in this age group undergo a major shift from concern for external things to their own bodies, the feeling of loneliness and isolation increases, and the intellect begins to diminish, which leads to emotional instability. Females are more prone to experience negative events and emotions, including postpartum mood swings, domestic violence, sexual abuse, and bullying, all of which may contribute to higher rates of depression^32–34^. Additionally, related reports have found that females are more selfless than males and are thus more prone to depression^35^. This finding partly explains sex differences in the onset of depression.

Our reports indicate that the rates of the two subtypes of depression, dysthymia and major depressive disorders, have remained largely stable globally and regionally over the study period, with the majority of patients suffering from MDD. The World Mental Health Survey estimated the annual prevalence of MDD to be 4.4% and the lifetime prevalence to be approximately 10% -15%^36,37^. With the continuous development of the social economy, accelerated pace of life, and increasingly fierce social competition, people’s psychological pressure is gradually increasing, and the incidence of MDD is increasing annually. MDD not only seriously affects the psychological condition and quality of life of patients, but also imposes tremendous mental and economic burdens to families and society^38^. In 2008, the WHO listed MDD as the third largest contributor to the global disease burden and predicted that MDD would be the leading contributor to the burden of disease by 2030^39^. Currently, about 300 million people worldwide suffer from MDD; therefore, effective control of major depressive disorders is an effective approach to preventing and managing depression^40^.

The findings suggest that Uganda’s relatively concentrated population and high prevalence of tropical diseases, malaria, AIDS, Ebola virus, sleeping sickness, viral hepatitis, and tuberculosis may be associated with its highest global ASR and ASDR for depression. Although the peak of the epidemic in Uganda, which had one of the world’s highest HIV prevalence rates, has passed and the rate of new cases has diminished in recent years, the number of people infected with the virus and living with the disease remains high, especially in rural areas. People living with HIV experience social prejudice and discrimination, which can lead to unemployment, poverty, family disintegration, and physical and psychological problems that can lead to low self-esteem, low mood, and even depression^41,42^. This shows that the prevention and control of HIV can affect depression.

The most significant rise in depression and DALYs was identified in Qatar, with the United Arab Emirates and Equatorial Guinea next. Significant increases in depression and DALYs were observed in the medium–high SDI and high-SDI regions. It could be because the level of economic development and education in these regions is relatively high, and the social pressure generated by residents is greater, leading to the increased prevalence of depression. Studies have found that individuals with different education levels have different levels of cognitive ability. Education level influences depression in individuals and can also impact spouses^43^. It is also true that social stress is an acknowledged risk factor for depression, and research have indicated that the higher the level of economic development, the more social stress people experience. Notably, the country with the most decline in depression and DALYs was Latvia, followed by Bosnia and Herzegovina, and Estonia^19^.

The ASIR and ASDR increased the most in Spain, followed by Mexico and Malaysia. These countries are reported to have higher economic incomes and sociodemographic indices, which confirm our statistics. However, in terms of ASR, the greatest decline in ASIR was in Singapore, then Sri Lanka and Slovenia; and the maximum decline in ASDR was in Singapore, Cuba, and Estonia^19^.

Further analysis of the relationship between illness and sociodemographic and geographic factors suggests that depression is more pronounced in terms of incidence in high-SDI and high-income countries, while the burden of depression is significantly higher in low-income and low-SDI countries.

In conclusion, the burden of depression varies across regions for a number of reasons. These include each region’s level of economic development, level of education, level of medical development and capacity to diagnose the illness, as well as the level of importance that governments attach to the illness^44–46^. Additionally, there are cultural differences that contribute to the different burdens of depression in different regions, such as customs and religious beliefs. The incidence of depression is still high, and the burden of the disease remains heavy, but the pathogenesis is unclear, which is an obstacle to the effective prevention and control of depression. This study nalysed the possible risk factors, such as domestic violence, bullying victimisation, and childhood sexual abuse. However, the GBD data show that the implication of these three risk factors on depression is small; that is, these three risk factors are not likely to be key risk factors for depression, indicating that this requires further investigation^47^.

To effectively prevent and control depression, governments must support depression-related research while taking appropriate steps to effectively address depression. For example, they should strengthen education on prevention and treatment, improve the capacity for early diagnosis and standardised treatment, establish mental health service measures for key populations, and carry out psychological intervention in a timely manner^19^.

This study performed the most comprehensive assessment of the depression burden to date. All the data used in this study were obtained from the GBD database, which offers a large sample size and high data quality, offering this study a distinct advantage in terms of data reliability. While numerous research have been conducted on the prevalence of depression in GBD 2019, the majority of these studies evaluate the condition using the age-period-cohort analytic approach, and their study regions, study objects, and focus subtypes of depression vary. Li et al., for instance, used the age-period cohort analytic approach to study the prevalence of depression among teenagers in the Western Pacific region^48^. In addition, Xu Y et al. used the age-period-cohort analytic method and limited their research to the incidence of depression across all areas, leaving unfinished business regarding studies on the burden of disorders like DALY^49^. Major depression is a subtype of depression, and Li et al. focused on examining gender variations in its illness burden. They discovered that women are more likely than males to experience major depression^31^. The disease burden of anxiety and major depression caused by bullying was examined by Hong C et al. as a risk factor. The findings indicated that from 1990 to 2019, there was a rising trend in both the DALY number and DALY rate of anxiety and major depression caused by bullying. Adopting effective techniques is necessary to eradicate bullying among children and adolescents^50^. Additionally, Yang F et al. used the age-period-cohort analysis method to conduct a thorough study on the prevalence of depression in 204 countries from 1990 to 2019. Findings from our study, such as the nations with the greatest and lowest incidence and DALY of depression, are in line with their conclusions. Moreover, the connection between the regional SDI and the depression incidence trend. But there isn’t any research on the prevalence and DALY of two distinct subtypes of depression, or on the connection between the HDI and depression burden, as well as the risk factors of depression^51^. This study comprehensively analysed the impact of depression and the temporal and spatial changes in disease burden at the global, 21 regional and 204 national levels. These include age-specific differences in the burden of depression, sex differences, and disease burden differences between the two subtypes of depression. Incidence, DALYs, ASIR, ASDR and EAPC (the change rate of ASIR and ASDR) are used to comprehensively describe the burden of disease from different perspectives and provide specific quantified values respectively. And most importantly, this study nalysed the relationship between the burden of depression and HDI, this is what is missing from all the relevant studies above. Finally, this study examined the depression risk factor data that was accessible in the GBD database. This will offer a crucial foundation for upcoming research on depression and its risk factors. All of the linked research mentioned above lack the analysis of risk variables. Nonetheless, this study possesses limitations. First, the GBD data used in this study were collected from a large amount of epidemiological survey data, processed, and integrated using corresponding statistical methods. The lack of original data in many countries and regions may have led to a bias in the differences in disease burden. Second, the comorbidity of MDD and dysthymia was excluded in this study. We hope that in the future the GBD database can further distinguish these disorders to facilitate a more complete analysis^52,53^.

## Conclusion

Depression remains a serious challenge worldwide, and its burden of disease remains heavy. By analysing the global burden of depression, this study clarifies the current situation of depression in various countries and provides a scientific reference basis for governments to formulate active and effective prevention and treatment strategies. Countries, especially those with a high burden of depression, must vigorously strengthen mental health education, actively prevent risk factors, and adopt targeted interventions to raise the level of awareness of depression among their populations, and concurrently, call for the reform of the relevant systems and the elimination of policy barriers to better prevent and treat mental health disorders^54,55^.

### Supplementary Information

Supplementary Information.

## Data Availability

The dataset generated for this study can be found in the GBD at http://ghdx.healthdata.org/gbd-results-tool.
