# Supplementary material for: Temporal and spatial trend analysis of all-cause depression burden based on Global Burden of Disease (GBD) 2019 study
Source: Sci Rep. 2024 May 29;14:12346. doi: 10.1038/s41598-024-62381-9 (PMC11137143; doi:10.1038/s41598-024-62381-9)
Supplement: Supplementary file 1 — Supplementary Information. [file 41598_2024_62381_MOESM1_ESM.pdf]

Supplements:

Fig S1 (Image generated in R software version 4.2.3 (https://cran.r-project.org))

a1 Oceania(ASIR)

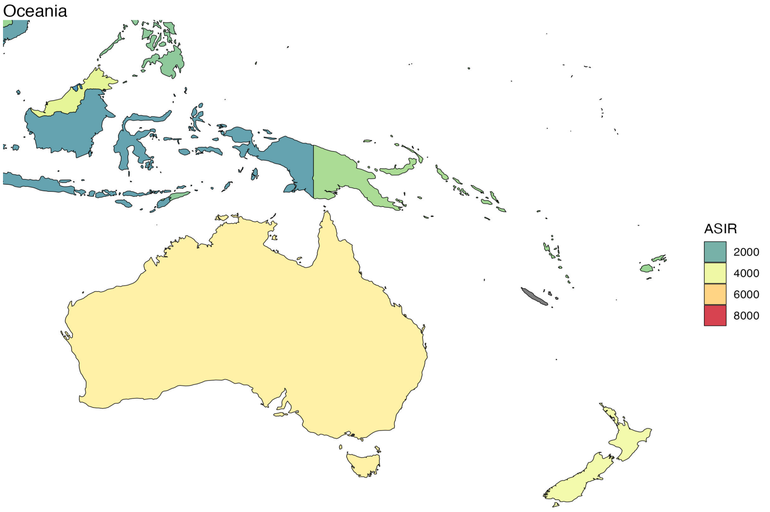

a2 Oceania(ASDR)

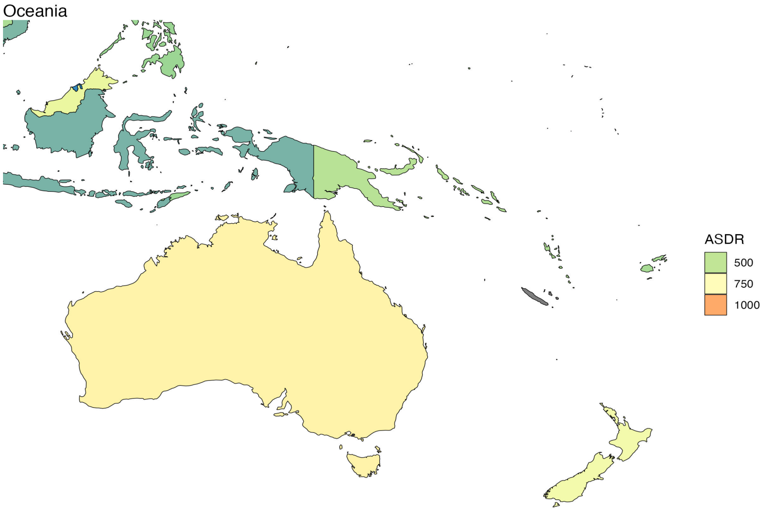

b1 Oceania (Incidence Change)

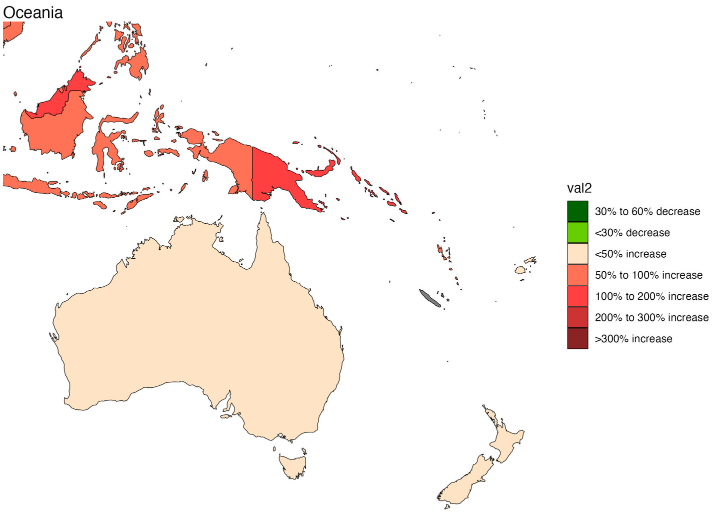

b2 Oceania (DALYs Change)

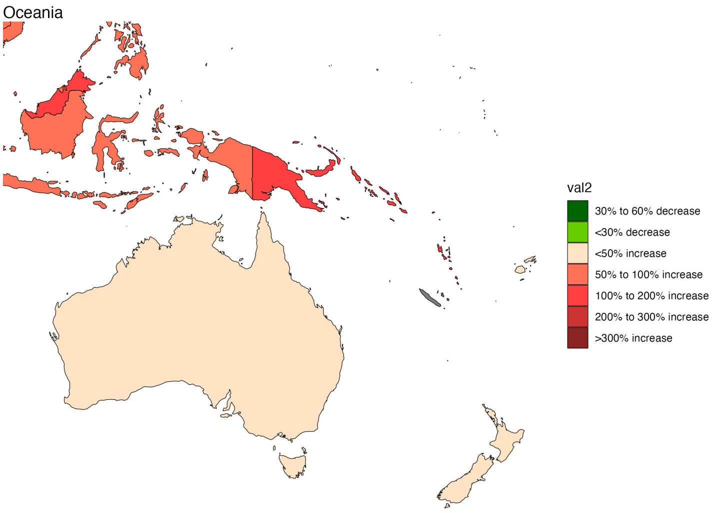

c1 Oceania (Incidence EAPC)

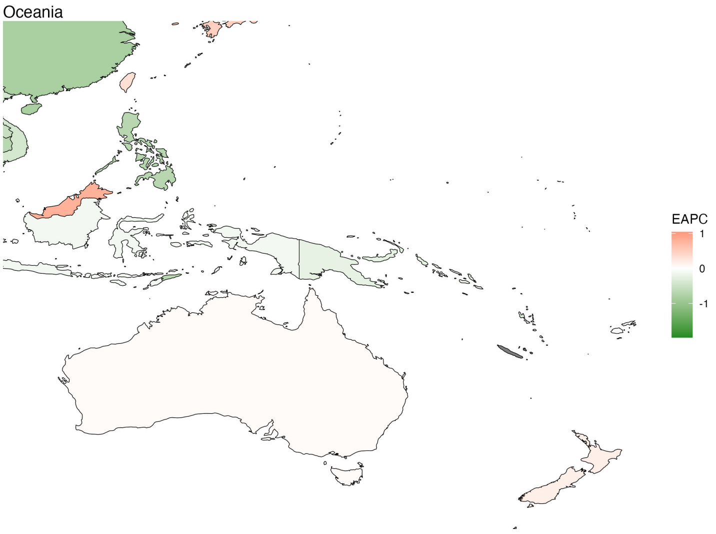

c2 Oceania (DALYs EAPC)

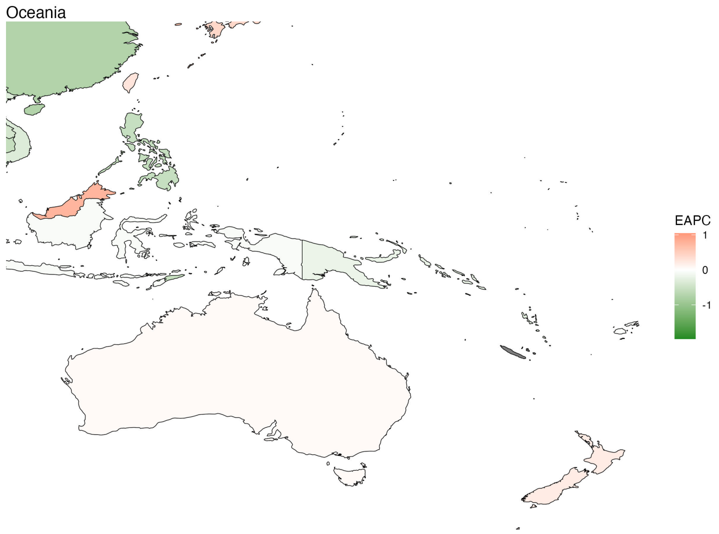

Table S1a. Incidence of Depressive disorders in 1990 and 2019 for Both sexes and 27 regions, with EAPC from 1990 and 2019.

location	Num_1990	ASR_1990	Num_2019	ASR_2019	Num_change	EAPC
Global	182183358 (159598111-207533227)	3681.24 (3239.27-4150.12)	290185742 (256024052-328260553)	3588.25 (3152.71-4060.42)	0.59% (0.55-0.64)	-0.29% (-0.38--0.21)
Sex						
Male	67157593 (58720942-76462995)	2735.94 (2415.75-3086.22)	110123422 (96668365-124305433)	2750.27 (2419.66-3104.07)	0.64% (0.6-0.68)	-0.21% (-0.3--0.11)
Female	115025765 (100839782-130899552)	4613.68 (4064.28-5203.44)	180062320 (159076846-204131417)	4416.34 (3886.9-5015.49)	0.57% (0.52-0.61)	-0.35% (-0.43--0.26)
Category						
Depressive disorders	182183358 (159598111-207533227)	3681.24 (3239.27-4150.12)	290185742 (256024052-328260553)	3588.25 (3152.71-4060.42)	0.59% (0.55-0.64)	-0.29% (-0.38--0.21)

location	Num_1990	ASR_1990	Num_2019	ASR_2019	Num_change	EAPC
Major depressive disorder	172720945 (150251619-197442629)	3494.53 (3075.98-3953.14)	274803790 (241280545-312774423)	3397.48 (2978.66-3866.97)	0.59% (0.54-0.64)	-0.31% (-0.41--0.22)
Dysthymia	9462413 (7777050-11415649)	186.7 (154.44-225.17)	15381951 (12782128-18474451)	190.77 (158.69-229.44)	0.63% (0.56-0.7)	0.08% (0.07-0.09)
Socio-demographic index						
High SDI	32612976 (29211108-36319475)	3647.38 (3259.76-4066.21)	44711792 (39796761-50166003)	4013.63 (3545.48-4550.43)	0.37% (0.33-0.4)	0.31% (0.18-0.44)
High-middle SDI	40601472 (35880545-45603060)	3482.47 (3092.5-3901.57)	53642569 (47529706-60307945)	3184.21 (2809.6-3583.66)	0.32% (0.27-0.37)	-0.5% (-0.57--0.43)
Middle SDI	49074918 (42578940-56180918)	3159.02 (2778.72-3577.15)	80760069 (71066732-91500542)	3139 (2765.35-3540.43)	0.61% (0.53-0.69)	-0.2% (-0.28--0.13)
Low-middle SDI	40830070 (35199537-47036548)	4517.61 (3941.27-5159.17)	70155480 (61292237-79973480)	4180.3 (3660.97-4740.48)	0.7% (0.65-0.76)	-0.62% (-0.79--0.44)
Low SDI	18957871 (16306041-22048722)	5040.65 (4383.63-5754.77)	40743981 (34959157-47317678)	4770.22 (4142.24-5461.66)	1.08% (1.05-1.1)	-0.38% (-0.5--0.26)
Region						

location	Num_1990	ASR_1990	Num_2019	ASR_2019	Num_change	EAPC
Andean Latin America	968879 (829998-1139536)	3064.13 (2663.53-3555.9)	1809802 (1561707-2084373)	2886.58 (2499.39-3315.19)	0.87% (0.76-0.98)	-0.29% (-0.34--0.25)
Australasia	1081675 (951541-1225348)	5029.45 (4423.34-5704.14)	1539866 (1329848-1771752)	5079.18 (4368.05-5925.8)	0.42% (0.32-0.53)	0.07% (-0.06-0.21)
Caribbean	1599511 (1372785-1855359)	4880.62 (4225.94-5632.55)	2156062 (1859121-2484561)	4336.17 (3737.42-5007.13)	0.35% (0.27-0.43)	-0.51% (-0.56--0.45)
Central Asia	2055713 (1778459-2362986)	3494.69 (3036.19-3992.04)	2980970 (2577906-3464935)	3327.42 (2888.66-3825.36)	0.45% (0.39-0.51)	-0.19% (-0.21--0.16)
Central Europe	3698533 (3259644-4184383)	2763.44 (2429.76-3118.42)	3557074 (3134283-4043451)	2436.8 (2132.45-2771.69)	-0.04% (-0.08-0.01)	-0.67% (-0.74--0.59)
Central Latin America	4454828 (3841324-5127165)	3351.05 (2941.55-3820.24)	9412732 (8221932-10719231)	3675.78 (3219.65-4181.96)	1.11% (1.03-1.2)	0.34% (0.3-0.37)
Central Sub-Saharan Africa	2798104 (2320781-3357243)	6893.34 (5875.09-8067.85)	6714339 (5590521-8062124)	6646.94 (5680.5-7819.84)	1.4% (1.29-1.51)	-0.17% (-0.18--0.15)
East Asia	32150710 (27847482-36663602)	2630.79 (2321.11-2971.91)	42235926 (37513979-47555038)	2292.26 (2043.67-2562.45)	0.31% (0.2-0.44)	-0.8% (-0.97--0.64)
Eastern Europe	9914671 (8568653-11312086)	3937.85 (3407.66-4509.38)	9150637 (7960749-10432658)	3546.8 (3076.08-4062.82)	-0.08% (-0.1--0.05)	-0.57% (-0.66--0.47)

location	Num_1990	ASR_1990	Num_2019	ASR_2019	Num_change	EAPC
Eastern Sub-Saharan Africa	7366572 (6309277-8559480)	5822.01 (5094.17-6646.88)	16013047 (13725944-18554113)	5466.48 (4781.02-6234.48)	1.17% (1.13-1.21)	-0.32% (-0.39--0.26)
High-income Asia Pacific	4229083 (3752097-4735370)	2216.42 (1970.63-2483.08)	5193652 (4652118-5735090)	2320.99 (2063.54-2600.8)	0.23% (0.17-0.29)	0.4% (0.27-0.53)
High-income North America	11368846 (10044209-12840355)	3805.68 (3369.31-4307.2)	18459876 (16429393-20674357)	4885.16 (4308.48-5532.44)	0.62% (0.59-0.66)	0.62% (0.32-0.92)
North Africa and Middle East	14584104 (12427442-17049279)	5068.92 (4380.9-5843.83)	31006695 (26270019-36438429)	5098.6 (4378.86-5947.72)	1.13% (1.03-1.23)	0.06% (0.03-0.09)
Oceania	158100 (131890-190665)	2810.22 (2388.36-3286.52)	328505 (274947-393381)	2711.59 (2306.26-3193.17)	1.08% (0.98-1.16)	-0.16% (-0.17--0.15)
South Asia	40418946 (34973493-46526519)	4653.99 (4057.19-5303.72)	71998403 (62917271-81675123)	4179.15 (3668.72-4727.18)	0.78% (0.72-0.84)	-0.85% (-1.1--0.6)
Southeast Asia	8944376 (7667914-10392270)	2151.87 (1877.32-2451.95)	14451056 (12506180-16471186)	2060.52 (1797.73-2341)	0.62% (0.54-0.69)	-0.19% (-0.25--0.14)
Southern Latin America	1770216 (1566158-2000056)	3648.77 (3228.45-4122.27)	2362146 (2089297-2658887)	3313.55 (2925.62-3745.45)	0.33% (0.27-0.4)	-0.42% (-0.5--0.34)
Southern Sub-Saharan Africa	1891774 (1635512-2153460)	4504.43 (3963.18-5083.59)	3344012 (2915269-3791826)	4552.32 (4015.91-5105.97)	0.77% (0.71-0.83)	0.13% (0.03-0.24)

location	Num_1990	ASR_1990	Num_2019	ASR_2019	Num_change	EAPC
Tropical Latin America	6679005 (5801500-7625987)	4873.33 (4268.6-5499.28)	10928342 (9746995-12123340)	4560.16 (4084.43-5058)	0.64% (0.55-0.72)	-0.32% (-0.62--0.02)
Western Europe	19718223 (17776852-21743431)	4536.45 (4082.49-5018.45)	22312186 (19873592-25015077)	4347.46 (3841.95-4912.74)	0.13% (0.09-0.17)	-0.11% (-0.14--0.08)
Western Sub-Saharan Africa	6331488 (5432312-7308919)	4690.81 (4077.69-5351.5)	14230414 (12217181-16431648)	4407.3 (3851.35-5021.82)	1.25% (1.22-1.28)	-0.25% (-0.43--0.07)

Table S1b. DALYs of Depressive disorder in 1990 and 2019 for Both Sexes and 27 regions, with EAPC from 1990 and 2019.

location	Num_1990	ASR_1990	Num_2019	ASR_2019	Num_change	EAPC
Global	29089267 (20346287-40034593)	588.57 (413.88-803.35)	46863642 (32929363-63797315)	577.75 (405.79-788.88)	0.61% (0.57-0.65)	-0.24% (-0.31--0.16)
Sex						
Male	11026254 (7679182-15201326)	449.36 (315.23-612.12)	18183102 (12682047-24947035)	452.17 (316.79-618.13)	0.65% (0.61-0.69)	-0.17% (-0.25--0.09)
Female	18063014 (12622020-24787892)	726.32 (510.43-988.59)	28680540 (20155773-39319358)	702.08 (492.3-963.58)	0.59% (0.54-0.63)	-0.28% (-0.35--0.21)
Category						

location	Num_1990	ASR_1990	Num_2019	ASR_2019	Num_change	EAPC
Depressive disorders	29089267 (20346287-40034593)	588.57 (413.88-803.35)	46863642 (32929363-63797315)	577.75 (405.79-788.88)	0.61% (0.57-0.65)	-0.24% (-0.31--0.16)
Major depressive disorder	23461280 (16026049-32502664)	472.98 (325.28-652.38)	37202742 (25650205-51217042)	459.59 (315.19-634.72)	0.59% (0.54-0.63)	-0.32% (-0.41--0.22)
Dysthymia	5627988 (3644258-8398941)	115.59 (75.42-169.77)	9660901 (6311566-14421787)	118.16 (77.31-176.65)	0.72% (0.66-0.77)	0.09% (0.08-0.1)
Socio-demographic index						
High SDI	5219254 (3674820-7061988)	581.59 (409.68-786.03)	7025129 (4955200-9506636)	626.84 (438.47-852.48)	0.35% (0.31-0.38)	0.23% (0.14-0.33)
High-middle SDI	6557856 (4607978-8899873)	560.68 (395.2-759.78)	8896917 (6247986-12123142)	523.01 (367.02-713.05)	0.36% (0.31-0.4)	-0.4% (-0.46--0.34)
Middle SDI	8102584 (5623130-11195418)	526.98 (370.29-720.45)	13541947 (9515935-18454507)	521.68 (366.8-709.93)	0.67% (0.59-0.75)	-0.18% (-0.24--0.13)
Low-middle SDI	6287261 (4351321-8642844)	696.11 (486.85-951.6)	11026538 (7715898-15191253)	654.34 (458.32-897.85)	0.75% (0.7-0.8)	-0.51% (-0.66--0.36)
Low SDI	2905699 (1979159-4020350)	769.74 (538.83-1052.44)	6345789 (4316623-8788145)	738.87 (514.68-1011.24)	1.18% (1.15-1.21)	-0.3% (-0.4--0.2)

location	Num_1990	ASR_1990	Num_2019	ASR_2019	Num_change	EAPC
Region						
Andean Latin America	153227 (103954-213870)	486.23 (336.58-677.58)	290671 (198567-403423)	462.07 (318.12-640.03)	0.9% (0.79-1)	-0.25% (-0.29--0.21)
Australasia	166228 (115025-225879)	769.93 (531.73-1047.67)	237564 (164169-330311)	777.82 (538.62-1094.07)	0.43% (0.33-0.53)	0.07% (-0.05-0.2)
Caribbean	241492 (164625-335405)	737.11 (503.44-1024.94)	327025 (226095-450595)	657.19 (454.07-905.93)	0.35% (0.28-0.43)	-0.49% (-0.54--0.43)
Central Asia	330100 (227567-462634)	557.38 (388.83-775.98)	486600 (334518-679840)	534.9 (372.57-741.82)	0.47% (0.42-0.53)	-0.16% (-0.18--0.13)
Central Europe	613247 (428683-841534)	458.39 (321.08-630.58)	596440 (420305-816082)	413.89 (290.54-572.31)	-0.03% (-0.07-0.01)	-0.54% (-0.6--0.48)
Central Latin America	689559 (475118-946220)	517.3 (361.59-709.24)	1447181 (1009408-1981151)	563.62 (392.72-771.17)	1.1% (1.02-1.18)	0.31% (0.28-0.34)
Central Sub-Saharan Africa	415927 (278639-587868)	1027.24 (701.08-1444.4)	1010267 (681633-1430655)	1000.16 (682.15-1397.69)	1.43% (1.33-1.54)	-0.12% (-0.14--0.11)
East Asia	5646651 (3941391-7772996)	468.39 (329.62-640.08)	7802555 (5472939-10767864)	415.98 (291.93-573.46)	0.38% (0.28-0.48)	-0.67% (-0.78--0.56)

location	Num_1990	ASR_1990	Num_2019	ASR_2019	Num_change	EAPC
Eastern Europe	1537853 (1079222-2114779)	611.06 (427.94-837.23)	1442695 (1013990-1986605)	562.24 (391.45-771.76)	-0.06% (-0.08--0.04)	-0.46% (-0.54--0.38)
Eastern Sub-Saharan Africa	1135041 (777007-1570224)	889.58 (620.16-1218.6)	2510165 (1702208-3475451)	845.4 (589.89-1154.93)	1.21% (1.17-1.25)	-0.26% (-0.32--0.21)
High-income Asia Pacific	674468 (472208-917321)	352.5 (247.02-477.98)	812255 (572741-1104805)	365.65 (253.57-499.26)	0.2% (0.15-0.26)	0.31% (0.2-0.43)
High-income North America	1877372 (1313241-2539730)	624.53 (437.14-847.68)	2864089 (2023936-3872481)	753.77 (525.53-1023.69)	0.53% (0.48-0.57)	0.43% (0.2-0.66)
North Africa and Middle East	2217457 (1509162-3064781)	777 (533.94-1064.69)	4767774 (3261470-6600677)	781.06 (535.18-1075.62)	1.15% (1.06-1.25)	0.06% (0.03-0.08)
Oceania	26650 (18019-37562)	490.87 (337.8-682.7)	56577 (38501-80206)	476.09 (325.58-663.22)	1.12% (1.03-1.21)	-0.13% (-0.13--0.12)
South Asia	6145367 (4259235-8413986)	704.15 (493.83-957.67)	11188435 (7828808-15283076)	645.08 (452.66-877.7)	0.82% (0.76-0.88)	-0.71% (-0.92--0.5)
Southeast Asia	1615661 (1104880-2257481)	400.64 (277.91-550.29)	2753223 (1898460-3795437)	389.23 (270.38-536.55)	0.7% (0.63-0.78)	-0.13% (-0.16--0.09)
Southern Latin America	268708 (188252-371394)	554.26 (388.51-766.27)	359571 (249695-491681)	503.29 (349.65-690.9)	0.34% (0.28-0.4)	-0.42% (-0.5--0.34)

location	Num_1990	ASR_1990	Num_2019	ASR_2019	Num_change	EAPC
Southern Sub-Saharan Africa	297417 (207003-411130)	704.67 (496.04-961.31)	524604 (368831-719717)	705.61 (497.87-958.57)	0.76% (0.71-0.81)	0.09% (-0.01-0.19)
Tropical Latin America	991317 (685443-1356868)	722.91 (502.48-984.74)	1652267 (1159774-2244114)	686.08 (482.44-932.46)	0.67% (0.59-0.75)	-0.27% (-0.54-0.01)
Western Europe	3054395 (2156475-4113050)	702.17 (491.63-950.74)	3463005 (2438349-4706017)	677.2 (475.01-929.5)	0.13% (0.09-0.17)	-0.09% (-0.11--0.06)
Western Sub-Saharan Africa	991132 (678812-1360913)	728.84 (507.96-997.72)	2270679 (1552645-3123065)	693.84 (485.18-949.29)	1.29% (1.26-1.32)	-0.2% (-0.36--0.04)

Table S2a.Incidence of Depressive disorders in 1990 and 2019 for both sexes in 204 countries,with EAPC from 1990 and 2019.

[illegible]	Num_1990	ASR_1990	Num_2019	ASR_2019	Num_c[illegible]
[illegible]an	544572 (459362-648409)	6046.05 (5129.58-7144.85)	1787825 (1480373-2138048)	5985.11 (5093.58-7009.94)	2.2[illegible] (2.05-[illegible]
[illegible]	62554 (53468-73842)	2106.36 (1826.64-2459.52)	71872 (62467-83246)	2221.47 (1927.99-2571.16)	0.1[illegible] (0.04-[illegible]
[illegible]	1021519 (854053-1226203)	4942.96 (4218.28-5823.45)	2007439 (1690524-2384297)	4763.39 (4043.84-5612.9)	0.9[illegible] (0.8-1[illegible]
[illegible]amoa	923 (773-1106)	2172.37 (1861.17-2523.49)	1168 (993-1375)	2123.05 (1821.6-2487.16)	0.2[illegible] (0.17-[illegible]
[illegible]	2463 (2100-2903)	4058.74 (3474.66-4779.73)	3973 (3358-4666)	3963.79 (3399.61-4673.08)	0.6[illegible] (0.48-[illegible]

	Num_1990	ASR_1990	Num_2019	ASR_2019	Num_c
	555660 (462020-671637)	7280.19 (6203.32-8625.91)	1530571 (1250687-1840483)	6951.37 (5841.51-8123.94)	1.7 (1.57-
arbuda	1936 (1625-2283)	3361.33 (2828.27-3949.2)	3362 (2823-3966)	3363.16 (2833.35-3953)	0.7 (0.62-
a	957171 (818516-1111436)	2957.88 (2529.73-3444.81)	1335229 (1189696-1485646)	2807.76 (2498.51-3136.48)	0.3 (0.28-
a	89070 (75399-103902)	2801.48 (2399.61-3243.75)	106860 (91257-124269)	3025.04 (2593.35-3497.92)	0.2 (0.1-
a	939022 (824341-1071175)	5244.4 (4591.23-6001.99)	1354889 (1160264-1575399)	5234.48 (4451.39-6152.63)	0.4 (0.32-
	350630 (301749-403793)	3996.18 (3433.46-4615.98)	341092 (296862-393078)	3240.07 (2804.95-3737.45)	-0.0 (-0.1-
an	173228 (146454-202918)	2694.25 (2312.92-3148.51)	289149 (245797-341623)	2676.21 (2295.47-3115.64)	0.6 (0.55-
s	8213 (6941-9670)	3466.24 (2974.08-4068)	14150 (11976-16632)	3412.99 (2909.49-3983.74)	0.7 (0.6-
	29900 (24747-35923)	6171.47 (5270.56-7195)	90981 (75710-108735)	5415.29 (4630.22-6335.16)	2.0 (1.68-
sh	4561069 (3826988-5384976)	5641.32 (4795.18-6623.02)	8475704 (7184241-9967015)	5423.44 (4620.14-6355.29)	0.8 (0.69-
s	9312 (8038-10911)	3536.64 (3038.4-4154.8)	12817 (10926-15084)	3589.01 (3071.48-4205.53)	0.3 (0.27-
	516803 (443587-597786)	4446.95 (3813.59-5139.53)	515608 (439029-593031)	4307.33 (3714.52-4949.6)	0 (-0.09-

	Num_1990	ASR_1990	Num_2019	ASR_2019	Num_c
ı	411217 (376967-447312)	3635.17 (3318.95-3963.92)	520028 (452093-600101)	3983.01 (3419.08-4617.41)	0.2 (0.15-
	5309 (4445-6213)	3636.97 (3125.36-4208.05)	14959 (12750-17610)	3787.34 (3256.93-4421.67)	1.8 (1.6-
	151349 (127540-178466)	4798.73 (4126.78-5600.68)	429176 (359766-505437)	4959.76 (4240.89-5737.42)	1.8 (1.6
a	2732 (2340-3178)	4198.35 (3604.4-4862.73)	2916 (2507-3389)	3592.12 (3090.39-4182.99)	0.0 (-0.04-
	21154 (17894-25185)	4899.22 (4210.43-5761.77)	33180 (28044-39330)	4537.95 (3870.54-5317.91)	0.5 (0.45-
ıal State of)	212907 (179642-250836)	4301.23 (3668.72-5034.11)	425749 (361490-494429)	3892.57 (3313.68-4500.57)	1 (0.87-
zegovina	161527 (138396-186566)	3438.57 (2948.55-3961.51)	113572 (96789-133867)	2674.07 (2289.43-3129.56)	-0. (-0.38-
a	42690 (36002-50436)	4507.71 (3832.6-5248.26)	99679 (83525-119525)	4655.87 (3987.62-5458.13)	1.3 (1.16-
	6540006 (5686095-7455955)	4893 (4290.16-5517.24)	10626932 (9481725-11782346)	4564.97 (4093.49-5060.95)	0.6 (0.54-
salam	3641 (3041-4369)	1571.85 (1353.18-1841.6)	7177 (6062-8505)	1575.58 (1346.16-1854.56)	0.9 (0.83-
ı	307612 (267262-354393)	3024.25 (2639.36-3478.52)	243971 (208548-280611)	2575.21 (2218.02-2984.95)	-0.2 (-0.28-
ıso	313495 (266612-367009)	4964.86 (4275.33-5728.64)	749773 (630823-878055)	4845.58 (4129.69-5603.59)	1.3 (1.26-

	Num_1990	ASR_1990	Num_2019	ASR_2019	Num_c
	243094<br>(203801-286200)	6500.26<br>(5532.9-7606.45)	432503<br>(363220-512953)	5240.03<br>(4501.88-6147.07)	0.78<br>(0.67-
de	13600<br>(11551-15914)	5160.39<br>(4392.4-5987.78)	29990<br>(25617-35079)	5567.46<br>(4775.72-6469.76)	1.21<br>(1.03-
a	241150<br>(200120-289762)	2951.64<br>(2508.62-3459.35)	407962<br>(348991-486828)	2529.96<br>(2177.8-2981.18)	0.69<br>(0.56-
n	358241<br>(300408-423320)	5020.03<br>(4302.1-5845.84)	1110145<br>(934989-1306362)	5204.11<br>(4503.56-6017.58)	2.1<br>(1.92-
	992996<br>(877028-1125508)	3378.05<br>(2975.64-3823.91)	1256284<br>(1102041-1428033)	3371.67<br>(2909.99-3888.79)	0.27<br>(0.19-
Republic	152882<br>(124926-183673)	7350.11<br>(6162.14-8638.51)	301327<br>(250174-360452)	7230.55<br>(6121.98-8465.86)	0.97<br>(0.86-
	236516<br>(199951-277478)	5838.4<br>(5028.99-6782.56)	613634<br>(514657-727275)	6002.42<br>(5148.67-6961.95)	1.59<br>(1.44-
	717863<br>(649450-790438)	5578.46<br>(5040.73-6137.07)	900684<br>(772238-1041824)	4516.87<br>(3872.09-5235.8)	0.25<br>(0.13-
	31303436<br>(27138796-35706054)	2647.72<br>(2335.5-2991.38)	41005280<br>(36457709-46160564)	2301.41<br>(2051.04-2570.6)	0.31<br>(0.19-
a	717958<br>(606629-844298)	2557.28<br>(2209.07-2998.44)	1161349<br>(1022646-1303790)	2265.12<br>(1997.96-2544.48)	0.62<br>(0.45-
s	15622<br>(13254-18505)	4791.08<br>(4119.63-5589.69)	28814<br>(24437-33971)	4565.68<br>(3898.29-5349.48)	0.84<br>(0.73-
	133189<br>(110949-159547)	7275.26<br>(6163.63-8532.67)	296992<br>(246507-356680)	6634.84<br>(5623.42-7818.33)	1.23<br>(1.07-

	Num_1990	ASR_1990	Num_2019	ASR_2019	Num_c
ıds	496 (396-619)	2833.97 (2300.2-3500.43)	547 (446-668)	2863.32 (2298.63-3557.47)	0.1 (0.02-
:a	90456 (77056-106062)	3502.71 (3033.98-4078.94)	188852 (161882-220280)	3692.1 (3174.84-4310.25)	1.0 (0.93-
ire	340186 (281967-407478)	4193.81 (3584.72-4878.73)	814228 (688463-952803)	4203.62 (3618.3-4876.99)	1.3 (1.25-
	210448 (182308-242768)	3727.46 (3241.73-4285.82)	174224 (150544-202454)	3043.22 (2630.72-3500.22)	-0.1 (-0.25-
	701764 (596492-824300)	6147.22 (5249.45-7225.74)	648715 (553818-750295)	4535.03 (3897.53-5253.04)	-0.0 (-0.18-
	25844 (21999-30433)	3216.62 (2744.08-3774.75)	47743 (40802-55946)	3214.22 (2742.79-3793.64)	0.8 (0.72-
	404118 (349995-462835)	3451.79 (3000.47-3981.17)	389524 (336597-448230)	2853.51 (2482.71-3262.53)	-0.0 (-0.12-
:public of Korea	478375 (406458-561128)	2425.06 (2088.78-2826.25)	642923 (554874-748831)	2129.83 (1843.5-2475.24)	0.3 (0.25-
of the Congo	1878656 (1555023-2265796)	6718.62 (5698.86-7889.42)	4401211 (3663423-5335233)	6507.69 (5544.14-7689.5)	1.3 (1.21-
	306186 (264734-355501)	5138.94 (4449.48-5978.1)	259048 (225921-297457)	3774.61 (3269.44-4341.86)	-0.1 (-0.23-
	15677 (12998-18726)	4861.76 (4170.72-5697.82)	49009 (40903-58283)	4888.47 (4179.99-5731.52)	2.1 (1.88-
a	2356 (2010-2747)	3426.25 (2929.81-4001.57)	2639 (2275-3101)	3452.56 (2964.95-4071.42)	0.1 (0.05-

	Num_1990	ASR_1990	Num_2019	ASR_2019	Num_c
public	268961<br>(225624-318144)	4479.06<br>(3814.72-5238.79)	488528<br>(415333-569506)	4562.94<br>(3902.04-5318.11)	0.8<br>(0.68-
	317019<br>(270181-378404)	3712.5<br>(3212.46-4307.91)	655708<br>(562717-766891)	3784.31<br>(3266.91-4423.02)	1.0<br>(0.89-
	2012810<br>(1689369-2392298)	4253.72<br>(3614.57-4997.06)	4170940<br>(3498985-4955670)	4487.85<br>(3816.95-5268.76)	1.0<br>(0.95-
or	197691<br>(169051-235510)	4431.31<br>(3815.06-5218.78)	250947<br>(214693-295662)	3975.09<br>(3414.66-4672.01)	0.2<br>(0.17-
uinea	23007<br>(19141-27468)	7433.64<br>(6273.27-8779.41)	78710<br>(64467-96495)	6941.01<br>(5864.84-8177.41)	2.4<br>(2.19-
	112522<br>(93683-134021)	5733.14<br>(4895.44-6693.11)	273506<br>(228943-324406)	5439.12<br>(4681.9-6342.01)	1.4<br>(1.28-
	99155<br>(85776-114061)	5593.35<br>(4830.08-6449)	72383<br>(61746-84290)	4113.96<br>(3562.12-4759.58)	-0.2<br>(-0.34-
i	24308<br>(20347-29148)	4477.99<br>(3814.72-5256.86)	46462<br>(39415-54994)	4900.23<br>(4196.66-5719.64)	0.9<br>(0.79-
	1977792<br>(1682827-2307255)	5818.33<br>(5016.74-6704.7)	3930137<br>(3345556-4595948)	5200.85<br>(4477.99-5987.28)	0.9<br>(0.95-
	17048<br>(14305-20287)	2494.13<br>(2128.64-2898.18)	22718<br>(19304-26610)	2513.68<br>(2146.54-2937.81)	0.3<br>(0.24-
	313743<br>(271771-365023)	5744.87<br>(4969.02-6712.15)	280753<br>(248688-319428)	4569.01<br>(3962.02-5261.45)	-0.1<br>(-0.19-
	3345438<br>(3063296-3626255)	5210.35<br>(4747.25-5663.48)	3281786<br>(2848376-3768110)	4302.02<br>(3687.31-4976.44)	-0.0<br>(-0.12-

	Num_1990	ASR_1990	Num_2019	ASR_2019	Num_c
	54709 (45811-65431)	6966.36 (5928.93-8173.8)	105527 (88007-126031)	6654.44 (5694.07-7805.39)	0.9 (0.81-
	43520 (36116-52213)	6630.35 (5722.5-7697.1)	108253 (90272-128988)	6582.28 (5651.08-7682.55)	1.4 (1.34-
	206697 (177907-239414)	3497.35 (3024.07-4037.34)	161207 (138973-187271)	3533.48 (3031.4-4089.6)	-0.2 (-0.27-
y	3176135 (2857605-3523766)	3422.97 (3071.51-3817.5)	3792182 (3309793-4349692)	3724 (3202.16-4305.42)	0.1 (0.09-
	511246 (431642-605267)	4820.72 (4120.04-5592.65)	1239552 (1046356-1452233)	4824.33 (4144.35-5617.74)	1.4 (1.3-1
	713076 (604277-831150)	6034.2 (5111.13-7035.42)	771810 (662195-899662)	6104.39 (5175.95-7171.73)	0.0 (0.01-
d	5029 (4197-6021)	8070.44 (6849.68-9510.42)	4205 (3528-4912)	7225.5 (6085.42-8458.12)	-0.1 (-0.23-
ι	2748 (2360-3200)	3673.77 (3161.05-4278.6)	4141 (3527-4848)	3677.83 (3149.61-4289.26)	0.5 (0.41-
	3965 (3313-4743)	2905 (2480.33-3392.58)	5117 (4393-5950)	2936.52 (2511.54-3412.43)	0.2 (0.19-
la	261357 (220687-308039)	4549.45 (3861.24-5329.86)	686600 (574260-806052)	4369.61 (3688.21-5088.64)	1.6 (1.47-
	206697 (174957-241233)	4639.2 (3958-5392.63)	430507 (364242-504837)	4844.88 (4133.9-5633.23)	1.0 (0.96-
sau	32801 (27549-38923)	4820.39 (4125.14-5654.78)	69056 (57763-82348)	5025.96 (4304.33-5825.98)	1.1 (0.99-

.	Num_1990	ASR_1990	Num_2019	ASR_2019	Num_c
	38459<br>(32369-45981)	5720.21<br>(4867.14-6751.8)	48454<br>(40917-57136)	6206.88<br>(5263.7-7271.32)	0.2<br>(0.16-
	226129<br>(189671-270213)	4407.85<br>(3761.65-5159.53)	475994<br>(395968-569720)	4271.1<br>(3628.79-5021.63)	1.1<br>(0.99-
s	113292<br>(95884-133360)	3439.39<br>(2963.65-3995.01)	305660<br>(259978-360884)	3616.26<br>(3117.21-4231.75)	1.7<br>(1.53-
/	439639<br>(382564-506421)	3651.03<br>(3174.63-4194.7)	360455<br>(313786-417657)	2770.17<br>(2415.41-3182.53)	-0.1<br>(-0.26-
	8879<br>(7694-10299)	3365.11<br>(2922.34-3902.28)	11281<br>(9826-12942)	2955.21<br>(2553.19-3427.95)	0.2<br>(0.17-
	31409144<br>(27155095-36290879)	4520.76<br>(3944.13-5161.57)	54175095<br>(47507666-61457139)	3975.68<br>(3497.85-4501.39)	0.7<br>(0.66-
a	3003071<br>(2550087-3519687)	1812.25<br>(1565.24-2078.77)	4862439<br>(4171102-5623268)	1794.07<br>(1557.18-2060.02)	0.6<br>(0.55-
public of)	2657707<br>(2180201-3195652)	5599.5<br>(4666.17-6625.94)	5346733<br>(4431342-6389394)	5895.48<br>(4929.12-6966.62)	1.0<br>(0.84-
	611245<br>(508438-730826)	4448.66<br>(3767.14-5232.58)	1785242<br>(1488963-2111559)	4465.11<br>(3802.93-5230.58)	1.9<br>(1.73-
	166584<br>(144939-191851)	4557.89<br>(3955.39-5253.59)	257423<br>(228754-288513)	4822.71<br>(4248-5511.65)	0.5<br>(0.43-
	245790<br>(212401-286887)	5097.06<br>(4409.32-5959.48)	448192<br>(386798-523310)	4724.39<br>(4068.5-5510.2)	0.8<br>(0.7-
	2818762<br>(2444128-3231482)	4218.49<br>(3622.68-4852.86)	2962207<br>(2586831-3360234)	3957.02<br>(3415.33-4581.61)	0.0<br>(0.01-

	Num_1990	ASR_1990	Num_2019	ASR_2019	Num_c
	71157 (59844-84357)	3370.18 (2850.87-3982.01)	103628 (88039-122422)	3420.27 (2908.96-4029.69)	0.4 (0.35-
	3183082 (2849827-3541572)	2248.72 (2005.95-2516.44)	3624190 (3253918-4006877)	2391.48 (2122.1-2693.71)	0.1 (0.09-
	159715 (130721-192385)	5415.26 (4569.5-6364.34)	536383 (447564-637950)	4819.98 (4084.46-5689.46)	2.3 (2.09-
an	570970 (492032-660782)	3797.87 (3294.68-4379.21)	682583 (589424-795678)	3709.14 (3212.98-4283.67)	0.2 (0.1-
	838629 (729346-958187)	5741.04 (5068.77-6484.03)	2035941 (1770232-2324373)	5307.04 (4709.1-5972.93)	1.4 (1.38-
	1830 (1504-2226)	2813.41 (2366.21-3327.28)	2803 (2359-3319)	2563.81 (2198.29-2992.92)	0.5 (0.43-
	75700 (61913-91697)	4492.34 (3795.81-5317.27)	233627 (192289-281879)	4677.72 (3950.1-5476.59)	2.0 (1.82-
an	149471 (128423-174272)	4026.34 (3493.06-4680.52)	214295 (181345-252635)	3621.31 (3091.6-4225.7)	0.4 (0.32-
atic Republic	86378 (72366-103340)	2531.26 (2149.54-2966.17)	156535 (131890-184083)	2244.92 (1925.74-2618.43)	0.8 (0.69-
	157009 (135433-183311)	5116.51 (4425.16-5937.93)	107239 (93247-121912)	4188.67 (3681.38-4764.16)	-0.3 (-0.38-
n	149648 (126530-176208)	5216.69 (4434.71-6123.69)	292831 (247993-343908)	5536.84 (4681.83-6501.7)	0.9 (0.78-
	89730 (75898-106062)	6435.49 (5472.51-7548.75)	122035 (103419-143461)	6548.41 (5663.51-7623.81)	0.3 (0.24-

	Num_1990	ASR_1990	Num_2019	ASR_2019	Num_c
	74338<br>(62863-87093)	5169.23<br>(4443.73-5999.2)	190470<br>(159298-225499)	5147.98<br>(4434.95-5966.09)	1.5<br>(1.4-1
	167783<br>(138771-202776)	4927.87<br>(4212.09-5844.03)	381775<br>(320704-453100)	5183.19<br>(4425.91-6142.7)	1.2<br>(1.09-
a	201141<br>(172712-233221)	4954.27<br>(4270.06-5749.36)	167428<br>(144219-194456)	4551.8<br>(3921.94-5270.04)	-0.1<br>(-0.24-
ırg	18619<br>(16044-21576)	4301.35<br>(3691.96-4994.99)	24178<br>(21287-27326)	3377.57<br>(2971.69-3854.36)	0.3<br>(0.18-
ar	451055<br>(379385-537515)	5482.95<br>(4660.07-6411.65)	1044437<br>(880426-1232697)	5283.04<br>(4529.61-6120.52)	1.3<br>(1.17-
	298484<br>(251951-353121)	4570.24<br>(3916.4-5325.55)	568739<br>(480240-671554)	4303.02<br>(3704.68-5007.66)	0.9<br>(0.79-
a	460808<br>(385597-542276)	3051.16<br>(2595.33-3543.05)	1169659<br>(1005970-1363474)	3619.1<br>(3131.46-4192.62)	1.5<br>(1.31-
s	5951<br>(4961-7064)	3470.82<br>(2980.16-4024.76)	13186<br>(11088-15559)	2572.33<br>(2205.67-2967.74)	1.2<br>(1-1.
	221900<br>(188218-261102)	3689.08<br>(3169.89-4298.39)	532821<br>(447199-633761)	3593.09<br>(3071.87-4200.3)	1.4<br>(1.24-
	13050<br>(11257-15154)	3325.78<br>(2861.73-3894.79)	16659<br>(14280-19332)	3246.27<br>(2764.42-3785.63)	0.2<br>(0.19-
ands	912<br>(752-1118)	2598.18<br>(2187.36-3065.56)	1345<br>(1123-1591)	2474.63<br>(2101.04-2892.32)	0.4<br>(0.37-
ia	57591<br>(48740-67646)	3920.45<br>(3334.02-4576.81)	116576<br>(98899-137876)	3760.99<br>(3232.56-4394.48)	1.0<br>(0.9-1

	Num_1990	ASR_1990	Num_2019	ASR_2019	Num_c
s	47867 (40701-57070)	4396.18 (3776.67-5155.53)	55635 (47581-64534)	3813.89 (3248.37-4439.07)	0.1 (0.06-
	2333864 (2031272-2641836)	3418.44 (3025.51-3836.32)	5358320 (4698444-6090644)	4159.23 (3662.48-4716.9)	1.3 (1.2-
ed States of)	2241 (1865-2715)	2669.7 (2286.64-3154.02)	2494 (2090-2972)	2498.09 (2114.04-2932.41)	0.1 (0.03-
	1725 (1421-2088)	4646.8 (3756.59-5701.53)	2100 (1715-2540)	4633.01 (3767.99-5698.46)	0.2 (0.15-
a	75891 (63630-89889)	4507.34 (3853.4-5254.67)	144203 (122183-169294)	4488.33 (3828.86-5217.39)	0.9 (0.73-
ro	18212 (15667-21128)	2827.58 (2438.64-3271.59)	20858 (17861-24142)	2761.02 (2363.55-3192.8)	0.1 (0.05-
	1370261 (1135760-1632129)	6341.63 (5366.53-7452.26)	2303008 (1944868-2736596)	6189.88 (5278.46-7322.91)	0.6 (0.55-
ue	494725 (417016-580725)	5406.39 (4640.36-6257.5)	1115448 (935620-1312248)	5645.46 (4828.56-6588.31)	1.2 (1.11-
r	508306 (428362-596917)	1415.5 (1213.05-1630.33)	772689 (655591-899072)	1393.92 (1188.1-1612.65)	0.5 (0.43-
	40131 (34239-47339)	3743.59 (3223.7-4367.17)	75908 (64671-89218)	3683.19 (3177.65-4257.17)	0.8 (0.77-
	235 (183-299)	2816.19 (2283.61-3468.48)	269 (211-343)	2845.86 (2312.51-3529.42)	0.1 (0.08-
	778029 (658034-916310)	5479.42 (4667.67-6359.98)	1617072 (1396697-1877765)	5836.38 (5047.85-6753.99)	1.0 (0.93-

	Num_1990	ASR_1990	Num_2019	ASR_2019	Num_c
ds	688869 (628787-747876)	4075.21 (3725.99-4432.41)	780178 (674896-906213)	3916.9 (3391.7-4554.76)	0.1 (0.03-
nd	142653 (118286-169721)	3955.96 (3288.69-4714.91)	184977 (160659-211772)	4187.43 (3593.01-4843.13)	0.3 (0.21-
a	106882 (89302-126692)	3702.4 (3150.31-4315.51)	231663 (196659-273108)	3755.14 (3236.51-4372.23)	1.1 (1-1.
	231144 (194457-274672)	4524.02 (3903.55-5245.89)	660261 (555634-779145)	4641.35 (3984.69-5391.07)	1.8 (1.69-
	3078976 (2641201-3571508)	4713.62 (4054.81-5442.68)	6026101 (5152836-7023851)	4025.57 (3497.8-4600.86)	0.9 (0.93-
	60 (49-74)	2849.65 (2314.58-3508.92)	50 (41-61)	2843.56 (2304.2-3532.19)	-0.1 (-0.22-
donia	52870 (45073-61582)	2603.14 (2237.54-3026.48)	62837 (53498-72675)	2394.98 (2053.15-2754.09)	0.1 (0.09-
a Islands	1012 (846-1220)	2185.69 (1881.24-2538.48)	1121 (958-1318)	2323.87 (1997.88-2708.68)	0.1 (-0.01-
	139143 (120784-160333)	2992.5 (2569.4-3453.01)	200791 (173283-230698)	3416.35 (2916.87-3955.87)	0.4 (0.41-
	72027 (59754-86662)	4544.17 (3869.46-5328.87)	218054 (177768-265834)	4584.86 (3908.84-5373.75)	2.0 (1.79-
	3649549 (3100156-4251534)	4588.14 (3909.32-5323.31)	7697352 (6516043-9019789)	4399.14 (3750.53-5080.11)	1.1 (1.05-
	425 (334-536)	2829.61 (2297-3500.72)	566 (456-696)	2805.81 (2271.86-3472.04)	0.3 (0.2-

	Num_1990	ASR_1990	Num_2019	ASR_2019	Num_c
e	121565<br>(102025-145618)	8000.35<br>(6792.27-9391.16)	342932<br>(287596-409853)	7864.2<br>(6719.71-9216.83)	1.8<br>(1.6
	69597<br>(58956-81759)	3342.71<br>(2858.25-3891.79)	138567<br>(118418-161181)	3290.39<br>(2812.1-3821.45)	0.9<br>(0.86-
uinea	103363<br>(85986-125040)	2916.06<br>(2478.38-3448.74)	245743<br>(204672-296636)	2756.74<br>(2347.03-3258.64)	1.3<br>(1.25-
y	139000<br>(116320-164866)	4044.95<br>(3454.09-4728.69)	301410<br>(255504-353603)	4353.14<br>(3740.79-5094.73)	1.1<br>(1.01-
	438953<br>(374234-518918)	2426.74<br>(2100.39-2824.09)	728345<br>(627914-847869)	2120.16<br>(1818.25-2460.6)	0.6<br>(0.51-
es	1480065<br>(1244556-1738475)	2824.34<br>(2430.19-3256.4)	2486763<br>(2117854-2879605)	2344.83<br>(2024.88-2692.8)	0.6<br>(0.64-
	788695<br>(682807-907776)	1952.98<br>(1691.42-2255.22)	925444<br>(804334-1055984)	1954.48<br>(1689.98-2263.28)	0.1<br>(0.13-
l	668782<br>(583207-779098)	5927.22<br>(5111.21-6914.83)	699727<br>(602910-813381)	5142.33<br>(4411.47-5976.62)	0.0<br>(-0.05-
co	119979<br>(103580-137617)	3287.59<br>(2838.7-3770.45)	136050<br>(116222-158054)	3141.81<br>(2691.28-3657.31)	0.1<br>(0.05-
	22500<br>(18436-27268)	4961.33<br>(4245.73-5808.92)	154919<br>(126544-189452)	4616.48<br>(3941.61-5428.45)	5.8<br>(5.34-
Korea	929542<br>(809561-1062928)	2043.51<br>(1795.18-2323.17)	1419063<br>(1262215-1581402)	2241.21<br>(1991.18-2513.99)	0.5<br>(0.38-
oldova	185387<br>(159465-215745)	4105.76<br>(3527.26-4762.31)	159388<br>(136363-188675)	3397.98<br>(2916.15-3983.6)	-0.1<br>(-0.22-

	Num_1990	ASR_1990	Num_2019	ASR_2019	Num_c
a	664944<br>(571408-771798)	2606.8<br>(2235.26-2993.03)	619718<br>(531724-715766)	2506.51<br>(2145.96-2865.8)	-0.0<br>(-0.15-
ration	5694224<br>(4916758-6570357)	3432.12<br>(2957.15-3963.3)	5594027<br>(4833375-6417230)	3183.18<br>(2749.78-3675.81)	-0.0<br>(-0.0
	327067<br>(274739-386613)	6842.89<br>(5862.04-7964.27)	576343<br>(486933-679307)	5818.52<br>(5007.58-6774.51)	0.7<br>(0.65-
l Nevis	1780<br>(1451-2196)	4760.91<br>(3882.13-5856.3)	3181<br>(2561-3936)	4723.08<br>(3843.24-5838.46)	0.7<br>(0.64-
ia	4389<br>(3709-5178)	3743.03<br>(3200.01-4381.5)	7580<br>(6496-8806)	3797.13<br>(3272.74-4409.76)	0.7<br>(0.59-
e Grenadines	3501<br>(2969-4110)	3713.92<br>(3187.45-4351.96)	4801<br>(4143-5631)	3874.14<br>(3349.49-4522.69)	0.3<br>(0.25-
	3731<br>(3089-4471)	2603.18<br>(2214.72-3058.77)	4635<br>(3925-5541)	2350.4<br>(2008.4-2768.91)	0.2<br>(0.17-
no	1211<br>(987-1483)	4625.14<br>(3771.45-5677.06)	1802<br>(1476-2186)	4681.85<br>(3791.73-5801.14)	0.4<br>(0.41-
Principe	3348<br>(2802-3988)	3813.35<br>(3242.21-4479.76)	6636<br>(5573-7881)	3850.26<br>(3294.63-4522.87)	0.9<br>(0.87
bia	610213<br>(505255-735639)	4555.99<br>(3872.37-5377.22)	1924143<br>(1593265-2316617)	4848.4<br>(4127.2-5680.67)	2.1<br>(1.81-
	218224<br>(185296-255801)	4286.27<br>(3686.82-4948.38)	495138<br>(418578-581910)	4330.55<br>(3711.8-5012.29)	1.2<br>(1.14-
	331427<br>(283661-384001)	3179.53<br>(2721.36-3661.16)	308067<br>(266370-356884)	2758.21<br>(2372.49-3182.05)	-0.0<br>(-0.16-

	Num_1990	ASR_1990	Num_2019	ASR_2019	Num_c
es	1612 (1374-1899)	2343.15 (1999.57-2715.06)	2352 (2022-2727)	2119.78 (1817.88-2470)	0.4 (0.34-
ne	123124 (104633-144991)	4556.64 (3912.62-5355.5)	303701 (256296-359587)	4886.12 (4180.95-5691.78)	1.4 (1.31-
e	112818 (98848-128934)	3465.61 (3062.32-3930.04)	143223 (124910-163012)	2264.45 (1968.76-2607.57)	0.2 (0.15
	163449 (142040-189956)	2918.3 (2542.73-3389.67)	177774 (152751-204898)	2577.16 (2229.32-2965.59)	0.0 (0.01-
	93038 (80932-106956)	4190.23 (3657.77-4826.34)	88756 (76851-101819)	3205.2 (2794.52-3688.96)	-0.0 (-0.14-
ands	7530 (6194-9071)	2780.78 (2367.43-3279.49)	15092 (12610-18140)	2646.2 (2249.86-3127.77)	1 (0.88-
	257777 (215592-307731)	5493.38 (4677.55-6441.33)	725829 (609416-856829)	5453.69 (4695.76-6375.94)	1.8 (1.6
ica	1473711 (1281212-1669007)	4729.35 (4162.7-5312.93)	2625283 (2303479-2958425)	4769.63 (4226.64-5322.73)	0.7 (0.72-
an	211311 (177306-251409)	5146.72 (4391.89-6017.85)	347893 (292255-412529)	5291.4 (4530.46-6174.36)	0.6 (0.54-
	1978602 (1813264-2146159)	4600.78 (4217.73-4992.26)	3078179 (2768359-3382751)	5570.03 (4949.52-6193.43)	0.5 (0.45-
a	535783 (456821-629280)	3182.32 (2746.34-3675.86)	557182 (482316-643545)	2412.36 (2083.65-2801.45)	0.0 (-0.04-
	860619 (709685-1040175)	5319.24 (4502.44-6312.38)	1863619 (1558103-2237029)	5201.33 (4401.68-6177.6)	1.1 (1.03-

	Num_1990	ASR_1990	Num_2019	ASR_2019	Num_c
e	19659 (16643-23186)	5526.19 (4707.81-6462.51)	35189 (29968-40784)	5850.97 (5013.28-6801.38)	0.7 (0.66-
	466170 (414414-525312)	4792.79 (4228.65-5440.15)	535507 (477354-601485)	4652.21 (4123.24-5276.28)	0.1 (0.08-
nd	395457 (343678-459300)	5054.31 (4395.05-5860.26)	413334 (367427-461517)	3978.53 (3520.25-4457.64)	0.0 (-0.04-
epublic	468728 (385659-564511)	4737.03 (4027.84-5539.79)	701840 (587583-828570)	4747.27 (4018.18-5598.94)	0.5 (0.38-
of China)	368898 (317408-425051)	1831.87 (1588.43-2097.6)	587722 (508498-680830)	1957.2 (1702.02-2255.45)	0.5 (0.45-
n	122260 (104442-144316)	3058.49 (2630.33-3566.8)	223004 (190582-263463)	2778.86 (2418.84-3215.21)	0.8 (0.71-
l	1340645 (1129357-1572721)	2391.66 (2043.18-2758.08)	2019978 (1738690-2364511)	2393.68 (2062.4-2771.36)	0.5 (0.34
ste	17718 (14849-21016)	2744.08 (2364.97-3183.8)	27729 (23306-33082)	2341.13 (2007.41-2718.69)	0.5 (0.47-
	114980 (95927-136981)	4848.22 (4097.63-5690.05)	304198 (256840-358656)	5034.25 (4301.69-5879.98)	1.6 (1.48-
	40 (32-50)	2865.05 (2332.56-3562.41)	37 (30-46)	2825.45 (2296.89-3506.74)	-0.0 (-0.11-
	1838 (1547-2197)	2201 (1882.31-2566.93)	2058 (1747-2425)	2169.17 (1861.91-2532.58)	0.1 (0.05-
Tobago	54003 (45420-64806)	4840.19 (4109.86-5729.12)	71200 (60764-83262)	4560.36 (3886.99-5307.24)	0.3 (0.18-

	Num_1990	ASR_1990	Num_2019	ASR_2019	Num_c
	448195 (374876-533674)	6154.56 (5235.42-7219.26)	765620 (647431-903096)	6138.57 (5181.93-7228.23)	0.7 (0.56-
	2520826 (2238825-2833264)	4641.15 (4155.41-5153.74)	4034113 (3445595-4739923)	4471.83 (3835.07-5226.36)	0.6 (0.45-
tan	97618 (83067-115189)	3413.55 (2935.18-3973.13)	149243 (127728-175710)	3129.09 (2680.13-3646.49)	0.5 (0.41-
	245 (195-305)	2870.27 (2319.42-3535.93)	334 (268-417)	2830.08 (2302.54-3528.96)	0.3 (0.31-
	932741 (777672-1115189)	8364.87 (7187.48-9854.47)	2188091 (1820741-2607449)	8062.76 (6946.5-9436.97)	1.3 (1.17-
	3060953 (2632987-3514687)	5021.06 (4309.79-5774.09)	2534563 (2176044-2910461)	4449.26 (3829.47-5141.73)	-0.1 (-0.21-
mirates	79035 (64704-95185)	4382.36 (3739.05-5148.89)	459539 (367667-571697)	4031.52 (3408.6-4782.79)	4.8 (4.09-
dom	3445476 (2994083-3942351)	5387.92 (4642.03-6211.94)	3562762 (3097781-4064661)	4669.12 (4032.52-5346.54)	0.0 (0.01-
f Tanzania	948590 (799850-1125575)	5397.89 (4594.86-6302.27)	2099600 (1767568-2467654)	5139.84 (4400.77-5931.78)	1.2 (1.09-
America	10370562 (9163103-11721397)	3849.98 (3401.99-4356.91)	17199094 (15313033-19228217)	5047.93 (4448.85-5704.27)	0.6 (0.62
gin Islands	3840 (3259-4527)	3714.27 (3177.2-4337.43)	4721 (4048-5531)	3853.11 (3295.53-4507.69)	0.2 (0.13-
	95111 (81843-110054)	2931.24 (2508.01-3410.23)	126113 (108863-145737)	3330.82 (2849.79-3869.16)	0.3 (0.23-

	Num_1990	ASR_1990	Num_2019	ASR_2019	Num_c
ın	570510 (485779-664279)	3531.95 (3032.4-4067.12)	1010425 (857350-1185713)	3293.66 (2838.82-3826.52)	0.7 (0.63-
ı	3457 (2873-4154)	2783.27 (2360.5-3276.95)	6895 (5750-8221)	2604.31 (2209.65-3068.72)	0.9 (0.88-
ı Republic of)	563731 (474314-667846)	3644.44 (3135.68-4252.53)	1090775 (932323-1275831)	3701.34 (3176.62-4320.2)	0.9 (0.78-
n	1203132 (1023950-1426278)	2037.8 (1753.88-2370.17)	1900015 (1615539-2210934)	1832.79 (1569.74-2122.41)	0.5 (0.46-
	569727 (468281-692950)	5854.24 (4897.22-6980.68)	1573630 (1290651-1890745)	5910.92 (4992.06-6992.41)	1.7 (1.6-1
	236080 (197277-280617)	4493.32 (3848.24-5248.35)	583956 (487477-697520)	4497 (3834.59-5246.63)	1.4 (1.32-
'e	221205 (185208-261120)	3231.21 (2771.91-3749.61)	374644 (316196-440932)	3351.17 (2893.18-3896.81)	0.6 (0.6-(

Table S2b. DALYs of Depressive disorders in 1990 and 2019 for both sexes in 204 countries,with EAPC from 1990 and 2019.

ion	Num_1990	ASR_1990	Num_2019	ASR_2019	Num_char
ıistan	79662 (54348-113050)	895.23 (611.17-1248.9)	264717 (177493-375779)	890.19 (608.04-1238.01)	2.32% (2.08-2.5
.nia	10998 (7468-15339)	369.02 (253.52-518.04)	12427 (8535-17238)	386.42 (266.46-536.31)	0.13% (0.03-0.2
ːria	156238 (104796-219776)	763 (520.54-1056.02)	312277 (210139-434502)	736.51 (500.15-1018.75)	1% (0.83-1.1

ion	Num_1990	ASR_1990	Num_2019	ASR_2019	Num_char
ı Samoa	165<br>(112-233)	402.72<br>(276.93-567.77)	214<br>(147-302)	393.94<br>(267.13-552.26)	0.3%<br>(0.22-0.3
ɔrra	393<br>(269-544)	640.44<br>(438.46-884.33)	631<br>(434-873)	626.03<br>(425.65-860.32)	0.6%<br>(0.48-0.7
ola	82982<br>(54760-117872)	1089.21<br>(745.07-1535.85)	230336<br>(155040-325500)	1046.88<br>(723.02-1466.33)	1.78%<br>(1.61-1.9
d Barbuda	302<br>(203-417)	524.82<br>(358.81-723.91)	527<br>(359-725)	524.06<br>(354.2-721.08)	0.74%<br>(0.63-0.8
ıtina	147438<br>(102362-203747)	456.59<br>(316.24-630.72)	204697<br>(142342-281628)	429.62<br>(297.35-591.33)	0.39%<br>(0.28-0.5
ɛnia	14839<br>(10019-20492)	460.92<br>(315.87-634.96)	17363<br>(11942-24081)	492.75<br>(342.64-679.56)	0.17%<br>(0.09-0.2
ralia	143733<br>(99094-194749)	799.47<br>(550.62-1084.7)	208113<br>(143255-290957)	798.72<br>(549.39-1128.52)	0.45%<br>(0.33-0.5
ria	55366<br>(38331-76689)	628.71<br>(431.86-874.27)	55068<br>(38830-76744)	523.26<br>(363.2-730.16)	-0.01%<br>(-0.08-0.0
aijan	29112<br>(19756-40581)	447.92<br>(309.08-615.87)	49028<br>(33343-68749)	445.11<br>(306.49-621.39)	0.68%<br>(0.58-0.8
mas	1285<br>(861-1791)	539.87<br>(368.29-753.2)	2214<br>(1499-3107)	531.32<br>(359.53-737.09)	0.72%<br>(0.59-0.8
ain	4564<br>(3067-6377)	932.78<br>(644.37-1294.62)	14098<br>(9452-19781)	823.25<br>(563.09-1149.98)	2.09%<br>(1.73-2.4
ıdesh	686483<br>(461500-947895)	850.62<br>(578.64-1183.15)	1289390<br>(884327-1794771)	822.2<br>(563-1140.18)	0.88%<br>(0.72-1.0

ion	Num_1990	ASR_1990	Num_2019	ASR_2019	Num_char
ıdos	1450<br>(989-2001)	550.99<br>(376.92-762.74)	1982<br>(1379-2768)	556.72<br>(382.44-772.19)	0.37%<br>(0.26-0.4
rus	79618<br>(55048-111526)	686.35<br>(473.62-958.95)	79351<br>(54538-109531)	668.09<br>(457.8-915.66)	0%<br>(-0.08-0.0
ıum	64590<br>(45476-87279)	570.22<br>(398.47-776.18)	80753<br>(55771-112592)	619.57<br>(425.08-861.78)	0.25%<br>(0.14-0.3
ze	817<br>(549-1148)	565.09<br>(384.32-783.4)	2310<br>(1566-3241)	583.75<br>(402.06-809.67)	1.83%<br>(1.64-2.0
ıin	23667<br>(15924-33042)	746.15<br>(515.17-1034.59)	67625<br>(45872-93642)	772.88<br>(532.8-1079.4)	1.86%<br>(1.71-2.0
ıuda	424<br>(290-594)	645.15<br>(444.14-904.81)	450<br>(313-635)	558.24<br>(383.67-775.75)	0.06%<br>(-0.03-0.1
tan	3242<br>(2194-4516)	744.32<br>(507.25-1042.61)	5144<br>(3524-7135)	696.86<br>(482.55-963.04)	0.59%<br>(0.49-0.7
tional State of)	32403<br>(21988-45175)	655.28<br>(449.51-912.53)	65958<br>(45423-91791)	600.07<br>(415.63-833.9)	1.04%<br>(0.91-1.1
Ierzegovina	26308<br>(18111-36363)	554.74<br>(385.46-764.83)	18669<br>(12770-25969)	445.31<br>(307.82-618.03)	-0.29%<br>(-0.36--0.:
vana	6734<br>(4546-9455)	708.9<br>(483.19-985.65)	15742<br>(10756-21790)	721.86<br>(497.28-993.68)	1.34%<br>(1.17-1.5
zil	970188<br>(671042-1327204)	725.33<br>(503.99-987.07)	1606200<br>(1128433-2183261)	686.56<br>(482.84-932.97)	0.66%<br>(0.57-0.7
russalam	612<br>(408-850)	259.03<br>(176.24-359.1)	1215<br>(825-1690)	260.29<br>(177.62-360.71)	0.99%<br>(0.84-1.1

ion	Num_1990	ASR_1990	Num_2019	ASR_2019	Num_char
aria	50004 (34350-69515)	494.37 (339.01-691.91)	39906 (27868-55377)	431.79 (299.53-601.6)	-0.2% (-0.27--0.[illegible]
Faso	48545 (33221-67808)	766.78 (527.74-1070.49)	118597 (79877-165051)	757.65 (514.68-1051.1)	1.44% (1.31-1.[illegible]
ndi	37271 (25052-52129)	988.3 (676.48-1378.07)	67833 (45487-95015)	811.36 (556.21-1125.79)	0.82% (0.71-0.9[illegible]
erde	2111 (1434-2924)	808.38 (556.55-1115.02)	4692 (3215-6586)	861.72 (590.63-1204.6)	1.22% (1.06-1.3[illegible]
odia	40438 (27170-56915)	514.18 (352.66-721.35)	73082 (50144-103020)	456.36 (314.5-643.72)	0.81% (0.67-0.9[illegible]
roon	55684 (37573-77705)	774.2 (534.17-1074.79)	173909 (117508-241141)	803.69 (552.31-1105.17)	2.12% (1.94-2.3[illegible]
ada	165208 (114622-225560)	556.9 (387.29-767.86)	210865 (145119-288620)	556.01 (381.5-763.12)	0.28% (0.2-0.3[illegible]
an Republic	22659 (14992-32178)	1089.95 (742.08-1515.43)	44962 (30343-64488)	1078.69 (740.25-1516.56)	0.98% (0.88-1.[illegible]
ad	36208 (24337-50384)	892.7 (612.74-1239.19)	93993 (62912-132480)	915.29 (628.18-1286.23)	1.6% (1.43-1.7[illegible]
le	106588 (74827-143768)	826.48 (575.96-1116.85)	135643 (93427-189073)	677.59 (461.51-941.62)	0.27% (0.15-0.4[illegible]
na	5486751 (3831876-7544138)	470.65 (330.97-643.29)	7561985 (5307782-10431737)	416.95 (292.97-574.39)	0.38% (0.28-0.4[illegible]
nbia	114480 (78685-159625)	406.02 (281.86-559.27)	188300 (130491-258858)	366.75 (252.97-504.4)	0.64% (0.48-0.[illegible]

ion	Num_1990	ASR_1990	Num_2019	ASR_2019	Num_char
oros	2471<br>(1670-3440)	754.12<br>(518.31-1049.36)	4628<br>(3159-6405)	724.82<br>(496.11-999.32)	0.87%<br>(0.76-1
go	19821<br>(13222-28252)	1087.83<br>(748.85-1524.72)	44991<br>(30397-63589)	999.48<br>(688.26-1400.73)	1.27%<br>(1.11-1.4
slands	85<br>(56-123)	497.32<br>(332.57-717.64)	97<br>(66-138)	500.37<br>(335.01-719.52)	0.14%<br>(0.05-0.2
Rica	14039<br>(9552-19341)	541.98<br>(374.35-748.52)	29189<br>(19934-40318)	568.76<br>(390.22-784.9)	1.08%<br>(0.92-1.2
Ivoire	54152<br>(36333-75577)	655.35<br>(452.27-909.64)	131407<br>(88951-181460)	664.33<br>(459.51-921.1)	1.43%<br>(1.28-1.5
atia	33337<br>(23211-46157)	590.72<br>(410.77-817.71)	27691<br>(19441-38058)	495.59<br>(343.31-683.13)	-0.17%<br>(-0.24--0.
oa	104845<br>(70443-148863)	916.26<br>(615.2-1279.5)	97323<br>(66782-135610)	684.92<br>(470.14-952.64)	-0.07%<br>(-0.17-0.
rus	4207<br>(2844-5776)	521.64<br>(353.79-715.25)	7832<br>(5415-11003)	521.45<br>(352.92-722.35)	0.86%<br>(0.75-0.9
chia	64325<br>(45180-89991)	552.52<br>(388.36-775.47)	63005<br>(44078-87311)	469.21<br>(324.67-647.75)	-0.02%<br>(-0.09-0.
Republic of Korea	88010<br>(60199-123572)	444.13<br>(302.95-617.15)	123289<br>(84353-173606)	401.11<br>(275.98-561.2)	0.4%<br>(0.32-0.
blic of the Congo	278888<br>(187311-394912)	1000.02<br>(681.17-1414.13)	662196<br>(441559-936456)	979.44<br>(665.47-1363.1)	1.37%<br>(1.24-1.5
nark	46901<br>(32103-65458)	787.03<br>(537.4-1095.83)	40802<br>(28445-55662)	597.1<br>(408.31-821.21)	-0.13%<br>(-0.2--0.0

ion	Num_1990	ASR_1990	Num_2019	ASR_2019	Num_char
outi	2513 (1677-3536)	764.33 (521.84-1060.47)	7900 (5296-10986)	769.7 (527.86-1068.62)	2.14% (1.92-2.3
nica	364 (247-501)	533.48 (365.75-741.97)	408 (282-563)	535.45 (368.08-740.73)	0.12% (0.06-0.
Republic	40884 (27657-57325)	681.46 (465.77-949.03)	74312 (50201-103857)	691.1 (466.13-966.43)	0.82% (0.68-0.9
dor	49199 (33189-69244)	579.08 (398.05-798.5)	102198 (70268-142186)	588.52 (404.92-816.24)	1.08% (0.91-1.2
pt	312797 (210950-435183)	664.78 (451.87-916.04)	648655 (434384-891781)	696.61 (472.23-956.27)	1.07% (0.95-1.
vador	29638 (20345-41931)	670.67 (463.42-942.46)	38317 (26470-53573)	607.14 (418.16-846.56)	0.29% (0.19-0.3
l Guinea	3394 (2278-4799)	1100.55 (748.88-1541.16)	11813 (7891-16821)	1041.59 (713.66-1462.05)	2.48% (2.26-2.7
rea	17378 (11677-24428)	874.38 (603.51-1219.95)	43078 (28916-59688)	840.27 (573.2-1169.08)	1.48% (1.33-1.6
nia	14928 (10368-20697)	844.55 (581.97-1178.24)	11024 (7551-15188)	639.98 (439.35-888.34)	-0.26% (-0.33--0.
tini	3854 (2608-5379)	706.88 (487.45-975.27)	7231 (4885-10177)	752.96 (516.51-1047.58)	0.88% (0.77-1
opia	305280 (208085-421135)	889.56 (618.03-1220.61)	619868 (419298-852564)	808.7 (562.26-1115.11)	1.03% (0.98-1.0
ji	2975 (2009-4150)	447.7 (305.2-625.63)	4070 (2787-5709)	448.39 (308.33-628.54)	0.37% (0.28-0.4

ion	Num_1990	ASR_1990	Num_2019	ASR_2019	Num_char
ınd	49108 (33512-67775)	892.3 (613.03-1235.16)	44574 (31161-61703)	722.9 (502.76-1007.18)	-0.09% (-0.17--0.[illegible]
ıce	512760 (364051-692778)	798.7 (566.31-1081.61)	511467 (353096-705360)	673.53 (460.82-934.88)	0% (-0.1-0.0[illegible]
on	8184 (5569-11473)	1045.67 (717.18-1455.92)	15971 (10873-22328)	1001.94 (681.8-1392.72)	0.95% (0.83-1.0
bia	6624 (4421-9250)	1002.37 (685.44-1393.14)	16491 (11045-22878)	994.19 (679.24-1381.09)	1.49% (1.35-1.6
·gia	33037 (22820-45952)	559.18 (386.77-774.4)	25198 (17485-35017)	561.36 (387.4-777.16)	-0.24% (-0.28--0.[illegible]
ıany	503881 (355063-686482)	542.21 (381.1-739)	591830 (410147-823105)	583.57 (403.49-814.67)	0.17% (0.08-0.2
na	80457 (53793-112366)	751.71 (515.79-1034.91)	196897 (131953-272547)	754.26 (515.73-1035.52)	1.45% (1.33-1.5
ece	108340 (73908-151286)	917.48 (620.19-1287.76)	116608 (80640-160647)	927.75 (626.5-1295.71)	0.08% (0.01-0.1
land	766 (515-1077)	1214.38 (825.48-1702.37)	647 (444-909)	1098.69 (750.76-1540.82)	-0.15% (-0.22--0.[illegible]
ada	421 (285-583)	568.19 (387.56-779.57)	641 (437-889)	567.08 (386.74-786.37)	0.52% (0.43-0.6
ım	685 (465-957)	509.99 (347.53-709.64)	903 (624-1266)	513.95 (356.04-723.95)	0.32% (0.22-0.4
mala	39067 (26483-54623)	680.84 (471.68-951.42)	104045 (70662-145969)	658.26 (450.76-919.84)	1.66% (1.51-1.8

ion	Num_1990	ASR_1990	Num_2019	ASR_2019	Num_char
ıea	32476 (22042-44585)	726.46 (503.68-997.57)	67700 (45517-94181)	756.12 (518.85-1046.94)	1.08% (0.97-1.2
Bissau	5140 (3438-7175)	750.35 (514.73-1053.74)	10883 (7306-15313)	780 (534.15-1082.26)	1.12% (1.01-1.2
ana	5681 (3831-8039)	845.65 (579.83-1173.8)	7174 (4848-10055)	915.44 (622.17-1272.22)	0.26% (0.16-0.3
iti	33952 (22885-47800)	664.44 (453.43-930.74)	72117 (48541-101994)	644.39 (442.06-893.06)	1.12% (1-1.24
uras	17414 (11679-24255)	528.84 (363.8-729.48)	47071 (31903-66372)	552.38 (375.66-763.12)	1.7% (1.55-1.8
gary	69454 (47718-97145)	579.93 (399.01-811.71)	58377 (40713-81252)	458.06 (316.03-634.13)	-0.16% (-0.23--0.(
ınd	1433 (978-1985)	542.61 (369.42-752.06)	1859 (1296-2569)	485.76 (334.79-674.41)	0.3% (0.2-0.4
ia	4778413 (3314056-6561829)	683.76 (481.92-929.27)	8456077 (5935019-11452573)	616.13 (433.49-834.61)	0.77% (0.7-0.8
ıesia	563510 (383818-782457)	351.41 (244.26-486.83)	956359 (657990-1333569)	350.26 (242.89-488.45)	0.7% (0.64-0.7
Republic of)	398047 (265359-558440)	848.12 (575.96-1182.04)	813441 (553621-1143612)	890.32 (605.75-1247.77)	1.04% (0.89-1.2
q	93102 (62911-129459)	686.5 (468.24-950.13)	275980 (187822-383412)	690.87 (476.11-965.21)	1.96% (1.78-2.1
ınd	25992 (18037-36115)	713.25 (495.31-988.68)	40067 (28259-54898)	749.2 (523.72-1033.65)	0.54% (0.44-0.6

ion	Num_1990	ASR_1990	Num_2019	ASR_2019	Num_char
el	37760 (25762-52987)	785.73 (537.41-1106.97)	69424 (48241-95469)	734.19 (506.39-1009.93)	0.84% (0.72-0.9
ly	434967 (303990-593281)	652.26 (452.85-891.7)	459748 (322897-627660)	620.38 (429.17-850.84)	0.06% (0.02-0.0
ica	11071 (7440-15362)	527.27 (359.79-729.9)	16167 (10978-22375)	532.13 (361.43-734.82)	0.46% (0.36-0.5
an	505358 (354367-689713)	356.73 (250.42-486.34)	561185 (397400-760555)	373.85 (260.7-510.79)	0.11% (0.07-0.1
lan	24146 (16098-34138)	826.33 (563.62-1136.39)	82815 (54895-114298)	743.53 (505.02-1027.87)	2.43% (2.16-2.7
hstan	90544 (62228-126514)	596.69 (413.41-833.17)	109154 (76174-152631)	586.6 (410.93-815.96)	0.21% (0.13-0.2
ya	129490 (89555-177853)	876.11 (613.11-1197.33)	320267 (223005-439831)	820.2 (574.45-1111.71)	1.47% (1.43-1.5
oati	310 (209-441)	491.16 (334.67-687.69)	492 (335-692)	457.48 (317.15-644.41)	0.59% (0.49-0.6
vait	11842 (7904-16733)	698.16 (475.93-963.98)	36780 (24630-51341)	723.95 (488.4-1001.48)	2.11% (1.86-2.4
zstan	23523 (16084-32768)	631.03 (433.87-876.8)	34639 (23676-48763)	577.77 (398.58-809.09)	0.47% (0.37-0.5
ocratic Republic	14966 (10140-21047)	456.4 (314.02-640.07)	28544 (19457-40477)	417.54 (287.05-590.16)	0.91% (0.79-1.0
via	23671 (16391-32546)	774.94 (537.31-1064.46)	16329 (11460-22457)	650.92 (453.75-893.62)	-0.31% (-0.37--0.2

ion	Num_1990	ASR_1990	Num_2019	ASR_2019	Num_char
non	22771 (15421-31806)	796.6 (543.93-1108.29)	44714 (30555-62306)	842.01 (576.15-1165.86)	0.96% (0.8-1.1
tho	13634 (9252-19025)	978.57 (672.44-1360.48)	18453 (12623-25684)	979.28 (676.03-1349.38)	0.35% (0.24-0.4
ria	11278 (7708-15577)	785.69 (545.5-1078.96)	29540 (19844-41023)	786.62 (541.06-1092.57)	1.62% (1.45-1.8
ya	25530 (17045-35768)	759.43 (517.69-1049.98)	58752 (40038-81749)	791.47 (540.3-1091.49)	1.3% (1.12-1.5
ania	30591 (20931-42657)	754.64 (516.19-1047.77)	25418 (17593-35481)	702.43 (482.09-985.03)	-0.17% (-0.24--0.
bourg	2926 (2024-4044)	671.73 (462.28-929.93)	3896 (2741-5292)	542.15 (374.55-740.04)	0.33% (0.23-0.4
ascar	70189 (47390-97859)	846.31 (583.6-1181.94)	165430 (111442-228661)	823.27 (565.95-1145.41)	1.36% (1.22-1.4
awi	47403 (32100-65337)	717.59 (494.31-985.21)	91765 (61446-127015)	684.91 (472.33-951.08)	0.94% (0.82-1.0
ysia	78452 (53033-110992)	527.01 (363.68-739.82)	198752 (135308-276771)	611.66 (417.41-853.92)	1.53% (1.34-1.7
ives	971 (660-1356)	587 (406.99-816.84)	2395 (1633-3369)	459.45 (317.74-648.23)	1.47% (1.24-1.
li	35874 (24337-49410)	592.87 (406.53-815.51)	87076 (58180-120635)	583.25 (399.22-809.8)	1.43% (1.28-1.5
lta	2116 (1450-2929)	537.16 (365.37-744.31)	2700 (1893-3724)	525.51 (356.84-723.9)	0.28% (0.2-0.3

ion	Num_1990	ASR_1990	Num_2019	ASR_2019	Num_char
Islands	154 (102-217)	462.4 (314.54-645.94)	238 (162-334)	442.73 (305.47-620.53)	0.55% (0.44-0.6
tania	9317 (6268-13023)	630.59 (433.92-875.83)	19032 (12704-26650)	609.08 (413.61-852.35)	1.04% (0.92-1.1
itius	7788 (5288-11019)	721.53 (495.71-1010.11)	9463 (6526-13232)	636.81 (438.29-894.12)	0.22% (0.11-0.3
ico	360914 (249945-491873)	526.64 (370.23-717.11)	814932 (568817-1108767)	630.58 (441.22-856.39)	1.26% (1.17-1.3
erated States of)	379 (257-533)	473.04 (324.29-660.99)	442 (303-624)	448.36 (307.72-632.33)	0.17% (0.09-0.2
aco	268 (186-378)	723.26 (482.99-1025.56)	324 (224-460)	719.15 (479.86-1020.1)	0.21% (0.15-0.2
golia	11804 (7963-16592)	702.23 (481.57-974.3)	22872 (15537-31882)	700.27 (480.9-973.1)	0.94% (0.78-1.1
negro	3031 (2088-4203)	468.8 (323.72-651.17)	3434 (2383-4800)	458.39 (317.03-639.18)	0.13% (0.05-0.2
occo	206001 (139141-288359)	957.83 (654.47-1339.3)	348797 (238375-491895)	934.58 (640.09-1313.79)	0.69% (0.56-0.8
bique	76533 (52100-105774)	830.83 (572.17-1144.47)	173044 (116282-242477)	865.36 (595.02-1200.7)	1.26% (1.12-1.[illegible]
mar	102850 (68445-143773)	299.06 (203.42-423.35)	165225 (112739-234441)	298.19 (203.44-421.71)	0.61% (0.51-0.[illegible]
ibia	6470 (4383-9071)	602.83 (416.65-839.18)	12366 (8412-17155)	592.73 (408.15-820.92)	0.91% (0.79-1.0

ion	Num_1990	ASR_1990	Num_2019	ASR_2019	Num_cha
ru	40 (26-57)	494.56 (329.1-705.7)	46 (30-66)	497.95 (332.78-716.49)	0.14% (0.08-0.2
al	117121 (79281-164208)	819.74 (559.66-1137.98)	243462 (167910-337550)	874.04 (603.14-1205.18)	1.08% (0.94-1.2
lands	107963 (75278-146193)	636.63 (447.75-862.04)	122681 (85293-170933)	616.81 (425.44-861.71)	0.14% (0.04-0.2
ealand	22495 (15281-31312)	622.5 (421.29-864.84)	29451 (20429-40077)	658.14 (448.19-898.93)	0.31% (0.23-0.
agua	16233 (10947-22837)	567.77 (394.09-783.48)	35789 (24412-49634)	576.93 (395.64-800.55)	1.2% (1.05-1.3
er	36568 (24375-51286)	710.78 (483.87-1000.75)	104298 (70322-146040)	730 (502.7-1013.5)	1.85% (1.69-2.0
eria	480370 (329041-660148)	729.85 (505.64-1001.84)	972458 (663770-1329625)	639.55 (446.61-873.42)	1.02% (0.99-1.0
ie	10 (7-15)	499.55 (337.52-717.9)	9 (6-13)	495.44 (331.5-705.64)	-0.15% (-0.2--0.
acedonia	8921 (6193-12534)	435.78 (304.07-609.21)	10675 (7446-14832)	406.8 (282.49-562.95)	0.2% (0.11-0.2
iana Islands	185 (124-261)	404.96 (276.98-566.08)	211 (145-298)	424.89 (292.68-590.75)	0.14% (0.03-0.2
vay	23037 (16131-31489)	493.01 (339.6-672.69)	32686 (22653-44556)	552.05 (377.98-757.12)	0.42% (0.39-0.4
an	11137 (7485-15494)	703.26 (476.86-972.04)	34444 (23132-48845)	707.8 (481.49-976.56)	2.09% (1.87-2.3

ion	Num_1990	ASR_1990	Num_2019	ASR_2019	Num_chai
stan	560108 (385002-777465)	702.87 (489.48-971.84)	1194362 (821108-1645724)	678.27 (474.27-937.43)	1.13% (1.07-1.2
au	73 (48-105)	496.08 (333.6-710.87)	102 (68-146)	488.66 (322.88-700.87)	0.39% (0.27-0.5
tine	17876 (11966-25444)	1189.65 (812.13-1673.48)	50911 (33940-71662)	1168.68 (802.95-1624.31)	1.85% (1.69-2.0
ma	10815 (7364-15087)	518.34 (357.32-719.21)	21528 (14800-29812)	510.99 (351.65-706.14)	0.99% (0.87-1.1
v Guinea	17259 (11614-24270)	504.7 (344.4-699.88)	42021 (28581-59502)	481.9 (329.39-673.24)	1.43% (1.3-1.5
guay	21129 (14147-29583)	620.25 (424.82-854.48)	46067 (31245-63754)	663.9 (450.77-914.69)	1.18% (1.03-1.3
u	71625 (48154-100201)	397.13 (270.34-552.36)	122515 (82703-170254)	355.22 (239.54-492.97)	0.71% (0.57-0.8
pines	249462 (169725-343289)	488.15 (339.04-677.1)	447204 (307303-617568)	424.69 (292.61-587.44)	0.79% (0.74-0.8
nd	140574 (98227-195044)	346.88 (242.69-481.25)	165228 (115351-229980)	349.13 (245.06-486.47)	0.18% (0.14-0.2
ugal	100995 (68577-139941)	896.76 (606.79-1251.42)	105981 (72594-145769)	787.15 (538.99-1080.81)	0.05% (-0.05-0.1
Rico	18761 (12937-25755)	514.64 (353.09-706.07)	21152 (14753-29469)	493.66 (337.54-684.74)	0.13% (0.05-0.2
ar	3495 (2321-4905)	760.15 (521.46-1044.81)	24435 (16199-34385)	708.38 (487.12-981.53)	5.99% (5.45-6.5

ion	Num_1990	ASR_1990	Num_2019	ASR_2019	Num_char
of Korea	151184 (102870-208818)	329.09 (227.69-453.28)	227039 (158456-308654)	357.64 (248.35-491.66)	0.5% (0.37-0.6
f Moldova	28808 (19686-39991)	635.98 (436.22-878.78)	25202 (17091-35472)	540.83 (366.7-763.79)	-0.13% (-0.2--0.0
ania	110957 (76209-155341)	436.44 (297.26-605.24)	103274 (71713-143406)	424.44 (293.42-593.3)	-0.07% (-0.14-0.0
ederation	901284 (630600-1233116)	542.32 (377.82-740.84)	899376 (632820-1234495)	513.08 (356.17-701.98)	0% (-0.02-0.0
nda	49838 (33615-70657)	1034.55 (710.15-1455.6)	89915 (59867-123812)	894.79 (614.38-1234.7)	0.8% (0.69-0.9
and Nevis	267 (176-384)	717.14 (479.77-1023.33)	481 (323-694)	711.29 (474.42-1012.72)	0.8% (0.67-0.9
Lucia	672 (451-928)	575.24 (394.28-790.82)	1167 (807-1595)	582.37 (401.74-796.9)	0.74% (0.6-0.8
l the Grenadines	537 (362-748)	573.27 (393.37-792.64)	737 (505-1027)	594.38 (404.79-828.01)	0.37% (0.26-0.4
noa	636 (432-896)	465.56 (320.03-653.86)	823 (562-1153)	428.03 (293.98-596.75)	0.29% (0.22-0.3
arino	189 (127-268)	719.82 (476.78-1032.06)	279 (191-393)	726.71 (489.03-1025.16)	0.48% (0.4-0.5
nd Principe	537 (357-752)	617.12 (420.69-862.56)	1082 (722-1503)	620.89 (423.1-859.8)	1.01% (0.9-1.1
Arabia	94640 (62231-133602)	709.39 (482.79-984.97)	302217 (202785-427149)	748.71 (515.68-1040.33)	2.19% (1.88-2.

ion	Num_1990	ASR_1990	Num_2019	ASR_2019	Num_char
gal	34660<br>(23315-48372)	676.25<br>(463-939.54)	79061<br>(53618-110937)	683.39<br>(470.97-950.49)	1.28%<br>(1.16-1.4
ia	53919<br>(37597-75022)	516.73<br>(359.27-717.79)	50052<br>(34646-69128)	456.63<br>(317.71-633.55)	-0.07%<br>(-0.15-0
elles	290<br>(196-406)	431.39<br>(297.02-602.85)	451<br>(310-641)	396.99<br>(271.5-560.88)	0.56%<br>(0.44-0.6
Leone	19365<br>(13061-26808)	712.42<br>(486.17-993.12)	47857<br>(32164-66866)	760.36<br>(523.96-1059.19)	1.47%<br>(1.32-1.6
pore	17313<br>(11861-23625)	524.55<br>(361.64-713.83)	22816<br>(15764-31379)	355.22<br>(243.19-490.02)	0.32%<br>(0.2-0.4
kia	26828<br>(18626-37402)	479.3<br>(332.74-667.85)	29600<br>(20729-40983)	432.43<br>(301.77-601.94)	0.1%<br>(0.03-0.1
nia	14589<br>(10038-20225)	656.83<br>(453.15-912.83)	14101<br>(9877-19511)	519.81<br>(360.85-715.81)	-0.03%<br>(-0.12-0.0
Islands	1260<br>(859-1788)	488.56<br>(333.85-679.89)	2603<br>(1773-3671)	468.57<br>(322.45-657.69)	1.07%<br>(0.94-1.2
alia	40042<br>(27260-55603)	846.28<br>(587.74-1178.41)	113651<br>(76970-157022)	842.25<br>(583.98-1174.03)	1.84%<br>(1.67-2.0
Africa	229924<br>(160454-315711)	734.48<br>(516.9-998.77)	408503<br>(286187-553756)	734<br>(518.5-995.8)	0.78%<br>(0.72-0.8
Sudan	32805<br>(22161-45438)	789.08<br>(544.19-1095.99)	53881<br>(36336-74292)	812.59<br>(561.03-1121.75)	0.64%<br>(0.54-0.7
in	309496<br>(216794-413905)	720.19<br>(505.01-967.31)	470096<br>(333377-640941)	854.2<br>(601.72-1168.32)	0.52%<br>(0.41-0.6

ion	Num_1990	ASR_1990	Num_2019	ASR_2019	Num_chai
ınka	90634 (62379-128260)	548.87 (379.39-771)	103567 (71048-145055)	440.87 (301.85-614.43)	0.14% (0.06-0.2
an	130278 (86978-182846)	815.34 (556.71-1123.54)	284188 (189631-398488)	797.86 (547.88-1109.1)	1.18% (1.04-1.3
ame	2941 (1991-4145)	827.33 (567.88-1158.2)	5239 (3583-7339)	869.93 (596.22-1217.88)	0.78% (0.65-0.9
den	72380 (50901-98388)	744.34 (520.95-1013.48)	83821 (58500-113874)	728.25 (506.36-986.92)	0.16% (0.1-0.21
rland	61381 (42675-84889)	780.5 (540.37-1085.51)	66093 (46581-89733)	635.31 (444.18-867.3)	0.08% (0-0.17
Republic	71488 (47706-100274)	733.55 (498.93-1010.88)	107651 (72315-148440)	731.25 (493.6-1010.88)	0.51% (0.39-0.6
nce of China)	71889 (48741-101342)	358.37 (244.46-502.65)	117280 (80813-165336)	378.76 (261-532.4)	0.63% (0.51-0.7
stan	19995 (13628-27746)	498.95 (345.31-690.42)	37632 (25323-52173)	459.08 (317.05-636.89)	0.88% (0.76-1.0
and	240253 (163728-343264)	436.77 (302.61-620.49)	379116 (259916-531693)	438.07 (298.87-612.27)	0.58% (0.43-0.7
Leste	3022 (2029-4260)	483.34 (331.19-673.7)	4890 (3273-6929)	427.71 (290.48-599.53)	0.62% (0.52-0.7
go	18067 (12041-25114)	755.69 (513.66-1044.67)	48051 (32714-66955)	783.33 (542.9-1084.01)	1.66% (1.5-1.8
lau	7 (5-10)	503.91 (337.48-725.16)	7 (4-9)	494.86 (335.2-711.52)	-0.05% (-0.1-0)

ion	Num_1990	ASR_1990	Num_2019	ASR_2019	Num_chai
ga	326 (223-459)	409.01 (279.16-578.66)	375 (256-527)	404.09 (277.9-569.46)	0.15% (0.09-0.2
ıd Tobago	8176 (5598-11572)	732.25 (502.79-1026.1)	10824 (7508-15024)	691.44 (475.77-963.63)	0.32% (0.2-0.4
sia	67855 (45698-95199)	933.97 (640.32-1284.62)	116559 (79616-163075)	930.15 (636.46-1292.58)	0.72% (0.58-0.8
‹ey	387518 (269793-529380)	717.21 (502.41-972.93)	632644 (435552-870791)	696.29 (477.3-952.56)	0.63% (0.49-0.
ınistan	15790 (10701-21959)	548.3 (375.73-767.47)	24638 (16911-33964)	508.89 (350.89-698.22)	0.56% (0.45-0.6
alu	43 (28-61)	504.47 (338.64-716.76)	58 (39-83)	494.93 (332.94-714.19)	0.36% (0.3-0.4
ında	138651 (93322-196632)	1237.97 (840.89-1725.15)	331453 (220857-468303)	1212.09 (824.6-1696.26)	1.39% (1.24-1.5
ine	458953 (319874-630961)	756.61 (529.74-1039.92)	385994 (270087-529791)	683.42 (475.7-942.98)	-0.16% (-0.2--0.1
ɔ Emirates	12414 (8206-17517)	680.05 (463.87-949.43)	73408 (48290-105239)	628.63 (425.93-866.17)	4.91% (4.24-5.6
ingdom	525408 (365938-714378)	819.9 (563.01-1123.67)	550766 (384308-753705)	721.29 (498.36-986.73)	0.05% (0.03-0.0
c of Tanzania	146811 (98989-203369)	830.45 (569.42-1166.04)	331617 (225826-461650)	802.27 (553.09-1112.22)	1.26% (1.14-1.
of America	1711355 (1197014-2318739)	631.62 (441.09-859.22)	2652532 (1865075-3590012)	774.97 (540.42-1056.28)	0.55% (0.5-0.6

ion	Num_1990	ASR_1990	Num_2019	ASR_2019	Num_char
/irgin Islands	597<br>(405-829)	576.11<br>(393.27-800.05)	721<br>(496-1006)	593.11<br>(405.15-827.68)	0.21%<br>(0.11-0.3
uay	14671<br>(10114-20349)	453.45<br>(313.21-631.32)	19213<br>(13431-26745)	508.79<br>(351.46-706.61)	0.31%<br>(0.22-0.4
istan	91456<br>(63240-127580)	563.32<br>(390.02-786.88)	166075<br>(112520-233517)	530.95<br>(361.44-746.01)	0.82%<br>(0.68-0.9
ıatu	582<br>(398-824)	487.53<br>(337.08-683.01)	1195<br>(817-1681)	462.56<br>(318.55-650.11)	1.05%<br>(0.94-1.1
rian Republic of)	86959<br>(58835-123104)	559.47<br>(385.27-787.54)	168010<br>(114934-235462)	568.57<br>(389.87-797.6)	0.93%<br>(0.78-1.0
Nam	220878<br>(148996-308242)	389.69<br>(264.97-544.62)	380567<br>(258903-533769)	360.23<br>(244.42-502.95)	0.72%<br>(0.59-0.8
ıen	84564<br>(55920-118036)	882.96<br>(596.45-1230)	235446<br>(157547-328116)	890.99<br>(607.6-1235.38)	1.78%<br>(1.62-1.9
bia	37532<br>(25043-51968)	708.3<br>(488.31-983.67)	93830<br>(63161-131639)	708.94<br>(486.66-994.93)	1.5%<br>(1.35-1.6
ıbwe	36801<br>(24611-50990)	530.82<br>(367.16-734.41)	62309<br>(42197-86772)	544.53<br>(379.56-755.93)	0.69%<br>(0.61-0.7
